# Changes in anxiety and depression during the COVID-19 pandemic in the European population: A meta-analysis of changes and associations with restriction policies

**DOI:** 10.1192/j.eurpsy.2023.2467

**Published:** 2023-10-26

**Authors:** Veeleah Lok, Hugo Sjöqvist, Anna Sidorchuk, Pär Flodin, Walter Osika, Michael Daly, Philip Hyland, Lars H. Andersen, Peter Fallesen, Marcelo C. Cabrera, Ann K.S. Knudsen, Karen Wetherall, Emily Widnall, Jenny M. Groarke, Cherie Armour, Christina Dalman, Anna-Clara Hollander, Maria Niemi

**Affiliations:** 1 Department of Global Public Health, Karolinska Institutet, Stockholm, Sweden; 2 Department of Clinical Neuroscience, Centre for Psychiatry Research, Karolinska Institutet, Stockholm, Sweden; 3 Region Stockholm, Stockholm Health Care Services, Stockholm, Sweden; 4 Department of Neurobiology, Care Sciences and Society, Karolinska Institutet, Stockholm, Sweden; 5 Department of Psychology, Maynooth University, Kildare, Ireland; 6 ROCKWOOL Foundation Research Unit, Hedehusene, Denmark; 7 Swedish Institute for Social Research, Stockholm University, Stockholm, Sweden; 8 Department of Disease Burden, Norwegian Institute of Public Health, Bergen, Norway; 9 Suicidal Behaviour Research Laboratory, School of Health and Wellbeing, University of Glasgow, Glasgow, UK; 10 Population Health Sciences, University of Bristol, Bristol, UK; 11 School of Psychology, University of Galway, Galway, Ireland; 12 School of Psychology, Queen’s University Belfast, Belfast, UK

**Keywords:** anxiety, COVID-19 restrictions, depression, meta-analysis, social distancing

## Abstract

**Background:**

Early studies of common mental disorders (CMDs) during the COVID-19 pandemic mainly report increases; however, more recent findings have been mixed. Also, studies assessing the effects of restriction measures on CMDs show varied results. The aim of this meta-analysis was to assess changes in levels of CMDs from pre-/early to during the pandemic and the effects of restriction policies in the European population.

**Methods:**

We searched for studies assessing both pre-pandemic and peri-pandemic self-reported emotional distress and symptoms of depression or anxiety among nationally/regionally representative samples in Europe and collected microdata from those studies. Estimates of corona containment index were related to changes in CMDs using random-effects meta-regression.

**Results:**

Our search strategy resulted in findings from 15 datasets drawn from 8 European countries being included in the meta-analysis. There was no evidence of change in the prevalence of emotional distress, anxiety, or depression from before to during the pandemic; but from early pandemic periods to later periods, there were significant decreases in emotional distress and anxiety. Increased school restrictions and social distancing were associated with small increases in self-reported emotional distress.

**Conclusions:**

Despite initial concerns of increased emotional distress and mental illness due to the COVID-19 pandemic, the results from this meta-analysis indicate that there was a decrease in emotional distress and no change in anxiety or depression in the general population in Europe. Overall, our findings support the importance of strong governance when implementing periodic and robust restriction measures to combat the spread of COVID-19.

## Introduction

At the wake of the COVID-19 pandemic, leading global and local health authorities [1–3] as well as the public [4] expressed worries about what the pandemic and the restrictions imposed to curb its spread may do to public mental health – based on experiences of previous pandemics [5–7]. COVID-19 restriction measures were implemented by governments to reduce spread of the virus and typically included restrictions on movement, gatherings, business operations, and school closures, as well as the promotion of social distancing and hygiene measures. “Social distancing” has been one of the more common restrictions [8] and has included mandates for individuals to self-isolate at home if experiencing COVID-19 symptoms. Other common COVID-19 restrictions have included bans on large gatherings; school closures; closures of “non-essential” places; and limiting contact with those who are at higher risk of developing severe COVID-19 [9]. Policies have varied in their breadth and duration across countries, from partial restrictions to complete stay-at-home-orders. They have been linked to negative impacts on society such as loneliness, worry, social isolation [10], as well as increased stresses imposed on children and adolescents due to school closures [11]. It has also been suggested that COVID-19 restrictions could trigger anxiety and depression through increased feelings of loneliness, heightened grief after bereavement as a result of not being able to visit the dying or attend memorial services, and the deprivation of personal liberties [12]. Indeed, a previous study of 15 countries, 10 of which were European, found a significant albeit small association between policy stringency and psychological distress [13].

Findings regarding the effects of the pandemic on mental health have been mixed. A global meta-analysis of 48 studies covering the first year of the COVID-19 pandemic found that population mobility and daily COVID-19 infection rates were associated with an approximately 25% increase in the prevalence of probable major depressive disorder and generalized anxiety disorder in 2020 compared to 2019 [12]. The study concluded that the consequences of the pandemic and restriction measures included short- and long-term impacts on rates of common mental disorders (CMDs) [12]. On the other hand, other reviews and meta-analyses assessing studies with pre-pandemic to during pandemic data found no changes in the prevalence of a broader range of mental disorders and symptoms when assessed after mid-2020, though there was an initial increase in the prevalence at the beginning of the pandemic [14, 15].

Observing changes in CMDs during the COVID-19 pandemic is insufficient to prove that the pandemic gave rise to such changes. It is crucial to examine the association between CMDs and COVID-19 restriction measures, particularly in European countries, which is a relatively unexplored area [16], and has to our knowledge not been assessed in European countries in particular. The varying types, intensities, and timing of restrictions, along with similar healthcare systems and demographics in Europe, offer a unique chance to investigate the relationship between restriction measures and mental health. A better understanding of these associations may strengthen evidence-based policymaking and safeguard European population mental health during any future public health emergencies.

This aims of this study were to [1] determine how the COVID-19 pandemic affected rates of self-reported emotional distress (from now on referred to as “mild CMD” or “mild anxiety/depression”) and anxiety and depression (from now on “severe CMD” or “severe anxiety/depression”) across Europe and [2] explore whether public health restriction policies were related to changes in the rates of mild and severe CMDs. The research questions to be specifically addressed were:

1. How did rates of self-reported mild and severe CMDs change in Europe from pre- and early pandemic to later on during the COVID-19?
2. Were changes different for mild and severe CMDs?
3. Were changes in the prevalence of self-reported mild and severe CMDs different between the sexes and different age groups?
4. Were social distancing and school restriction measures associated with changes in self-reported mild and severe CMDs in Europe?

## Methods

The screening of the articles and reporting of this meta-analysis were guided by the Preferred Reporting Items for Systematic Reviews and Meta-Analyses (PRISMA) guidelines [17] and the Meta-analysis of Observational Studies in Epidemiology recommendations [18].

### Eligibility criteria

This meta-analysis included population-based prospective and cohort studies that addressed COVID-19 and CMD outcomes. Studies reporting the following data were included: (1) self-reported depression or/and anxiety; (2) depression or/and anxiety from pre-/early pandemic and during the COVID-19 pandemic; (3) data from any European country; (4) longitudinal or repeated cross-sectional studies; (5) study samples representative of the general population; and (6) the outcomes(s) measured by one if the validated instruments used in the previously published meta-analysis (e.g., GAD-7, HADS, PHQ-9, World Health Organization [WHO]-5 – for a comprehensive list, see Supplementary Table S2) [12]. Studies were excluded if they were editorial papers or cross-sectional studies that evaluated depression or anxiety at only one time point. No language limitations were set for the search, and both published studies and unpublished studies were included if data were available. No manual searches were conducted.

#### Timing of the COVID-19 pandemic

According to the WHO, COVID-19 could be characterized as a pandemic on March 11, 2020, and the countries included in this meta-analysis started implementing government restrictions spanning from March 10 (Czech Republic) to March 23 (UK). This study considered pre-, early, and during pandemic time waves, to allow for the study of changes in CMDs, as well as the impact of restriction measures, which in many cases had not been implemented in the early pandemic period of the present study: The pre-pandemic time wave of the present study refers to the first data collection time point before March 11, 2020, and the early pandemic wave refers to the first data collection point between March 11, 2020 ,and March 31, 2020 (apart from in Austria, where data were collected in April 2020). The pandemic time wave refers to the latest data collection point of each individual study during the pandemic – April 21, 2022 at latest. The latest time point was included to allow for the assessment of more enduring effects.

### Information sources and search strategy

This study builds upon previous findings from a meta-analysis on the effects of the COVID-19 pandemic on mental disorders [12]. The present study builds upon the search strategy and search results of the published study, including all the studies found through that review, as well as an additional literature search – therefore, the present meta-analysis includes studies from January 1, 2020 until April 21, 2022. The added value of the present study in relation to the previous meta-analysis [12] is the updated literature search, the narrower focus on European countries as detailed in the introduction, the assessment of the effects of restriction measures in particular, and assessment of effects on both mild and severe CMDs.

The searches for the present review were conducted in PubMed database (National Library of Medicine) (published studies) and in the COVID-MINDS database (gray literature). We included studies/protocols published between January 30, 2021 (which was the end point date of the previous meta-analysis search [12]), and April 21, 2022. The keywords used in the search included different COVID-19 terms, CMDs, European countries’ names, and CMD outcome measurements; the search strings are shown in Supplementary File S1. The self-report outcome measures and the cut-off thresholds that were used can be found in Supplementary File S2.

### Study selection

Two investigators (M.N. and V.L.) screened and identified potentially relevant titles, abstracts, and full texts independently. If there was any disagreement between the assessors, they consulted with a third investigator (P.F.) to reach an agreement. The Newcastle–Ottawa scale (NOS) was used to assess the quality of studies [19].

### Data extraction

A standardized data extraction template specifying requested data in detail was sent to all corresponding authors with access to microdata or aggregated data, depending on availability. The requested data included the following: the time period for the data collection, region, age group, sex, outcome measure, mean, 95% confidence interval (CI), standard deviation, number of respondents, number of respondents above mild threshold of self-reported anxiety and/or depression, and number of respondents above severe threshold of depression and/or anxiety. An invitation e-mail and three extra reminders in case of non-response were sent to all identified corresponding authors of the included studies.

The “female ratio” and “mean age” at baseline were collected from the original publications and datasets, and age ranges of 0–18, 19–64, and 65+ were used. The containment severity index was provided by Kubinec et al. [20] and measures the intensity of government responses to COVID-19 across six distinct policy areas (for more details, see Supplementary File S3). We only used the “social distancing” and “school closures” measures as independent variables. The choice was motivated by the direct cost of these particular policies on the social isolation of the people under a government’s jurisdiction, which may therefore incur impacts on CMDs.

For the independent variables data, we chose the point prevalence of CMD for each study based on their primary outcome pre-/early and during pandemic time frames. For both pre-/early and during pandemic time frames, the recall period of the outcome measure used in each study was added to the time frame. The recall period for each outcome measure is listed in Supplementary File S2. The mean value restriction measure of each pre- and during pandemic time frame was calculated to assess associations with the changes in the prevalence of CMDs. The difference in values was calculated as the data from the during pandemic time point minus the data from the pre-pandemic time point.

### Statistical analysis

The analyses generated pooled estimates of changes in the CMD prevalence in European countries both for (1) pre-during pandemic periods and for (2) early–during pandemic periods separately. Thus, the pooled results are reported as the average pre-/early-to-during difference in the prevalence (ΔPrev) and its 95% CI. Following the methodological recommendations [21], we also estimated and reported prediction intervals that reflect the uncertainty expected in the pooled results if a new study is included in the meta-analysis. We acknowledge that in case of high heterogeneity, the prediction interval will be wider than the 95% CI of the pooled results and that prediction intervals are imprecise if less than five studies are pooled [21] – thus, we do not report prediction intervals for subgroup analyses with <5 studies. First, we conducted a meta-analysis of changes in the prevalence of mild and/or severe anxiety and/or depression using random-effects analysis. Then, we studied those changes in mild and severe depression and anxiety in relation to demographic factors and changes in the COVID-19 restriction measures (in univariate meta-regression models, which were followed by additional adjustment for study quality). We ran models for the joint data of depressive disorder and anxiety disorders (mild and severe analyzed separately) first and then ran them separately for depressive disorders and anxiety disorders (mild and severe analyzed separately).

Statistical heterogeneity among studies was assessed using *Q*-test and *I* ^2^ statistics. For the Q statistic, a two-sided *P* < 0.10 was considered as representative of statistically significant heterogeneity, and *I* ^2^ values of 25, 50, and 75% were regarded as low, moderate, and high heterogeneity, respectively. Then, we conducted a univariate meta-regression relating changes in the prevalence of CMDs to changes in the restriction measures. Potential publication bias for each outcome in the main analyses was visualized by funnel plots and assessed using Egger regression asymmetry test [22]. If publication bias was revealed, the contour-enhanced funnel plot and Duval and Tweedie nonparametric trim and fill method were used to further test the data [23]. Sensitivity analyses were also conducted, where data from studies including children only [24] were excluded. Finally, influence analyses (also called “leave-one-out” analyses) were performed for each outcome by iteratively removing one study at a time to explore if the findings were influenced by any single study. All the analysis was performed in Stata MP 17.1 (StataCorp LLC, College Station, TX), and a two-sided *P*-value of < 0.05 was considered statistically significant. The codes for statistical analysis can be found in Supplementary Appendix 2.

## Results

### Study characteristics

**Figure 1.**
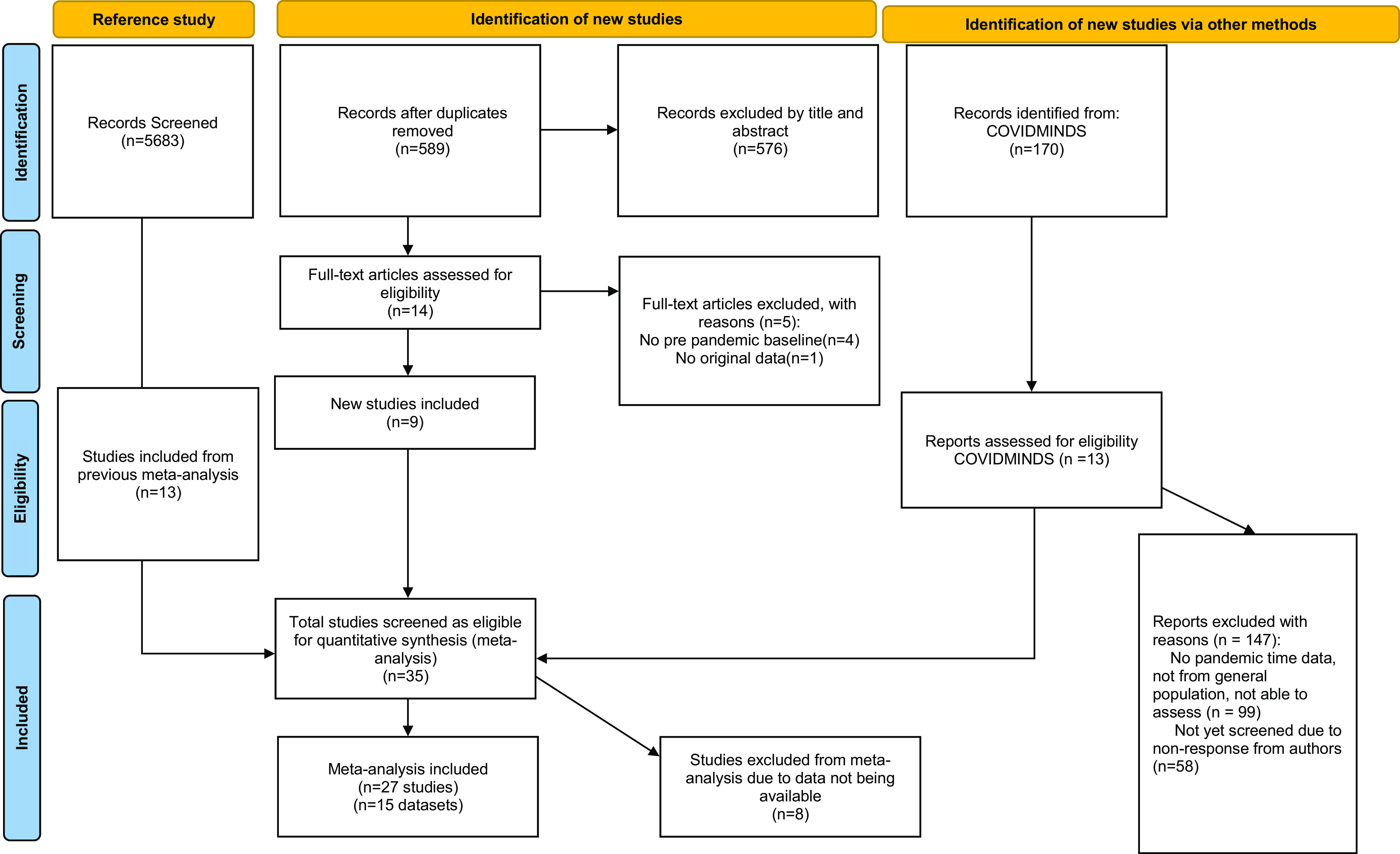
PRISMA flow diagram illustrating the study selection process.

The flowchart of the study selection process is shown in Figure 1. First, we included 13 studies from the previous meta-analysis (“Reference study” in Figure 1), which had screened altogether 5683 titles [12]. Thereafter, our PubMed search yielded a total of 589 additional titles of published articles that were screened for eligibility. After the screening of titles and abstracts, 576 articles were excluded, which resulted in 14 full texts being screened. Five of these were excluded, which resulted in nine published articles being included in the meta-analysis. The gray literature search yielded a total of 170 studies from title screening, and the further screening steps of contacting authors to gain information about the study data resulted in 13 studies being included from the gray literature screening. However, due to time and financial restrictions, we were only able to include the studies in the present meta-analysis for which authors were able to provide data by September 31, 2022, which resulted in the exclusion of 8 of the 13 studies from the gray literature screening. In total, our study selection process resulted in 15 datasets from 27 studies that were eligible for inclusion in this meta-analysis.

Table 1 overviews the included datasets and studies, and the total number of respondents at baseline from all studies was 88,620. The age range was from 12 to 99 years, the proportion of females was 54.7%, and the data of the included studies were gathered between October 2017 and March 2022. Studies with early pandemic data were from Spain [25], the United Kingdom [10, 26–30], Ireland [28, 29, 31], Denmark [32–34], and Austria [35, 36], and those with pre-pandemic data were from the Netherlands [37], the United Kingdom [24, 38–41], Ireland [42], Norway [43], and the Czech Republic [44, 45]. For a table including all results from all meta-analyses, please see Supplementary Tables S5 and S6, and for data visualizations in time plot graphs see Supplementary Figures S1 and S2. Of the 15 included studies, 11 obtained an overall quality score or 6 stars, while 4 obtained an overall quality score of 7 stars according to the NOS [19]. The quality scoring for each included study can be found in the Supplementary Table S4.

**Table 1.** Summary of studies (*n* = 27) and datasets (*n* = 15) included in the meta-analysis

First author	Source	Setting	Representativeness	Outcome	Instrument	Female ratio	Response rate	Age range	Time period		*N*, T2	Prevalence pre/early (%)		Prevalence during	
									Pre-/early	Pandemic		Mild	Severe	Mild	Severe
Ayuso-Mateos	PubMed	Spain	Provinces of Madrid and Barcelona (random sampling)	Depression	CIDI	0.60	Pre-/early: 68% Pandemic: N/A	18–75+	Jun 19–Mar 20	May–June 20	1104	9.0	0.0	7.9	4.0
Armour	Covid Minds	United Kingdom	National	Anxiety	GAD-7	0.50	Overall: 79.57%	0–90+	Mar 20	May 20	1566	30.5	15.3	22.9	10.1
				Depression	PHQ-9							34.5	17.1	30.8	14.6
Daly	PubMed	Ireland	National	Anxiety	GAD-7	0.57	N/A	18–65+	Mar 20	Dec 20	1092	20	8.4	22.3	8.9
				Depression	PHQ-9							22.7	11.0	29.1	16.6
Andersen	PubMed	Denmark	National (random sampling)	Depression	WHO5	0.55	Pre-/early: 34% Pandemic: 54%	18–90+	Mar 20	Dec 20	1628	19.4	3.7	15.9	0.4
O’Connor	PubMed/Covid Minds	United Kingdom	National (quota sampling)	Anxiety	GAD-7	0.54	Overall: 63.1%	18–75+	Mar 20	May 20	2601	21	12.7	16.8	7.0
				Depression	PHQ-9							21	8	23.7	11.2
Pieh	PubMed	Austria	National	Anxiety	GAD-7	0.53	Overall: 43.5%	18–65+	Apr 20	Sep 20	437	19	6	15.6	5.7
				Depression	PHQ-9							21	8.4	19.7	8.5
Hyland	Covid Minds	United Kingdom	National (quota sampling)	Anxiety	GAD-7	0.51	N/A	15–95+	Mar 20	Nov 21	1400	21.6	8.5	17.2	6.7
				Depression	PHQ-9							22	10.9	20.1	10.0
		Ireland	National (quota sampling)	Anxiety	GAD-7	0.52	N/A	15–95+	Mar 20	Mar 21	1105	22	8.4	18.1	7.1
				Depression	PHQ-9							22.8	11	23.1	11.6
Sonderskov	PubMed	Demark	National	Depression	WHO5	0.48	Overall: 48.67%	18–89	Mar 20	Dec 21	1428	25.9	4.5	21.2	5.9
Fancourt	Covid Minds	United Kingdom	National (snowball)	Anxiety	GAD-7	0.51	N/A	18–60+	Mar 20	Mar 22	11340	21.8	9.3	15.7	6.6
				Depression	PHQ-9							28.3	13.1	22.1	9.9
Knudsen	PubMed	Norway	Trondheim (probability sampling)	Anxiety	CIDI	0.54	Overall : 30.8%	20–65	Jan–Mar 20	Jun–Sep 20	774	–	9.1	–	8.5
				Depression	CIDI	0.54	Overall : 30.8%	20–65	Jan–Mar 20	Jun–Sep 20	774	–	2.5	–	2.7
Van der Velden	PubMed	Netherlands	National (probability sampling)	Depression and anxiety symptoms	MHI-5	0.51	N/A	18–65+	Nov 19	Jun 20	4084	16.8	6.3	15.3	5.1
Widnall	PubMed	United Kingdom	National	Depression	HADS	0.59	N/A	10–14	Oct 19	May 20	844	20.1	6.9	21.9	6.9
				Anxiety	HADS							49.1	29.4	41.7	23.8
Winkler	PubMed	Czech Republic	National (random sampling)	Anxiety	MINI	0.52	Overall : 75%	18–90+	Oct–Nov 17	Nov 20	2999	–	3.2	–	5.3
				Depression			Overall : 75%					–	4.0	–	12.1
UKHLS	PubMed	United Kingdom	National (probability sample)	Depression	GHQ-12	0.56	Pre-/early: 46% Pandemic: 48.6%	17–90+	Nov 19 –Mar 20	Sep 21	12806	31.6	20.2	28.6	19.4
Briggs	Covid Minds	Ireland	National (random sampling)	Depression	CESD-8	0.56	Overall: 71%	58–80+	Jan–Dec 18	Jul–Dec 20	3349	9.2	–	19.8	–

### Changes in the prevalence of mild and severe levels of anxiety and depression

**Figure 2.**
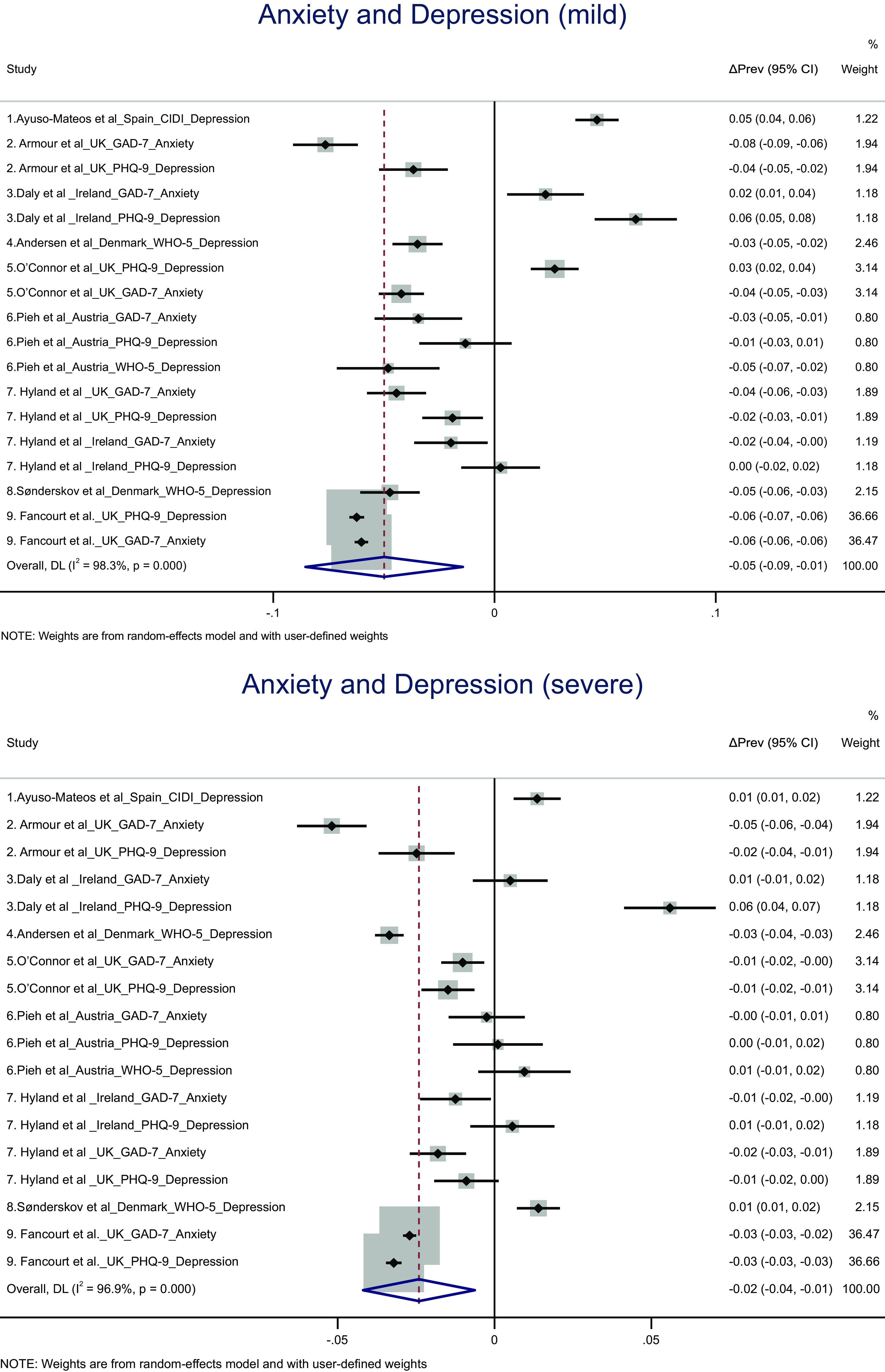
(A) Forest plot of random-effects meta-analysis results on early to during-pandemic changes in the prevalence of mild anxiety and depression. (B) Forest plot of random-effects meta-analysis results on early to during-pandemic changes in the prevalence of severe anxiety and depression. The results of individual studies and the pooled results are reported as the average early-to-during difference in the prevalence (ΔPrev) and its 95% confidence intervals (95% CI).

The meta-analysis of all included five datasets with data from pre-pandemic periods did not show any significant change in self-reported severe anxiety and/or depression from before the pandemic to during the pandemic in any of the demographic groups studied (see Supplementary Table S5) – the results did not significantly change in the sensitivity analyses where the dataset including children only [24] was removed (data not shown). Due to small number of studies, the analysis of publication bias and influence analysis were conducted only for anxiety and depression combined (mild and severe separately). No publication bias was detected (see Supplementary Figure S3B,C,E,F for corresponding funnel plots), and no influence of individual study on a pooled results was observed (see Supplementary Note S5). However, among the datasets with data from early pandemic, significant, but very minor decreases were found in levels of mild anxiety and depression combined (ΔPrev = −0.050 [95% CI: −0.085, −0.014], *P* = 0.006; *I* ^2^ for heterogeneity 98.3%; and 95% prediction interval [−0.13, 0.03]) (Figure 2), mild anxiety alone (ΔPrev = −0.055 [95% CI: −0.094, −0.017], *P* = 0.005; *I* ^2^ = 95.3%; and 95% prediction interval [−0.137, 0.026]), severe anxiety and depression combined (ΔPrev = −0.024 [95% CI: −0.042, −0.006], *P* = 0.008, *I* ^2^ = 96.9%, and 95% prediction interval [−0.066, 0.017]) (Figure 2B), and severe anxiety alone (ΔPrev = −0.025 [95% CI: −0.047, −0.003], *P* = 0.025, *I* ^2^ = 93.3%, and 95% prediction interval [−0.071, 0.021]) (see Supplementary Table S6). As reported in Supplementary Figure S3D,F, in the analyses of data from early pandemic, potential publication bias was detected for severe anxiety and depression combined and severe depression alone (Egger’s test *P*-value = 0.001 and 0.011, respectively; and trim-and-fill analysis results also suggested that the asymmetry in the corresponding funnel plots was likely related to publication bias). No potential publication bias was seen for other outcomes. Influence analyses revealed no indication that individual study, if omitted, would significantly influence the observed pooled estimates (see Supplementary Note S5).

### Changes in the prevalence of mild and severe anxiety and depression stratified by age and sex

None of the analyzed age and sex groups displayed significant pre–during pandemic changes in the prevalence of mild or severe depression or anxiety (combined or alone) (see Supplementary Table S6), and the results remained the same in the analysis when the dataset with children only [24] was removed (data not shown). Among studies with data from early pandemic, there were minor, but significant changes in the prevalence from early to during pandemic among the following age and sex groups: Among 19–64-year-olds, there was a significant decrease in mild depression and anxiety combined (ΔPrev = −0.059 (95% CI: −0.106, −0.013), *P* = 0.012; *I* ^2^ = 98.6%; and 95% prediction interval [−0.171, 0.052]). The significant decreases in mild depression and anxiety combined were found among males (ΔPrev = −0.039 [95% CI: −0.074, −0.004], *P* = 0.029; *I* ^2^ = 96.7%; and 95% prediction interval [−0.119, 0.041]) as well as females (ΔPrev = −0.059 (95% CI: −0.099, −0.019), *P* = 0.004; *I* ^2^ = 97.33%; and 95% prediction interval [−0.154, 0.035]), as was the decrease in mild anxiety among males (ΔPrev = −0.039 [95% CI: −0.073, −0.004], *P* = 0.029; *I* ^2^ = 89.9%; and 95% prediction interval [−0.111, 0.034]) and among females (ΔPrev = −0.070 (95% CI: −0.112, −0.028), *P* = 0.001; *I* ^2^ = 92.4%; and 95% prediction interval [−0.160, 0.020]). In addition, females in age group 65+ also reported a significant decrease in mild anxiety and depression combined (ΔPrev = −0.037[95% CI: −0.069, −0.005], *P* = 0.023; *I* ^2^ = 89.6%; and 95% prediction interval [−0.111, 0.036]) (see Supplementary Table S9). Moreover, with regard to severe depression and anxiety combined, there were significant decreases from early to during pandemic among 19–64-year-olds (ΔPrev = −0.031 [95% CI: −0.057, −0.005], *P* = 0.020; *I* ^2^ = 97.8%; and 95% prediction interval [−0.094, 0.032]) and among females (ΔPrev = −0.031 [95% CI: −0.051, −0.012], *P* = 0.002; *I* ^2^ = 94.6%; and 95% prediction interval [−0.078, 0.015]). Also, there were significant decreases in severe depression among females aged 0–18 (ΔPrev = 0.173 [95% CI: −0.325, −0.021], *P* = 0.026; *I* ^2^ = 88.4%; no prediction interval measures due to small number of studies). For detailed results, see Supplementary Tables S7–S10.

### Changes in the prevalence of mild and severe anxiety and depression from pre to during pandemic stratified by country

**Figure 3.**
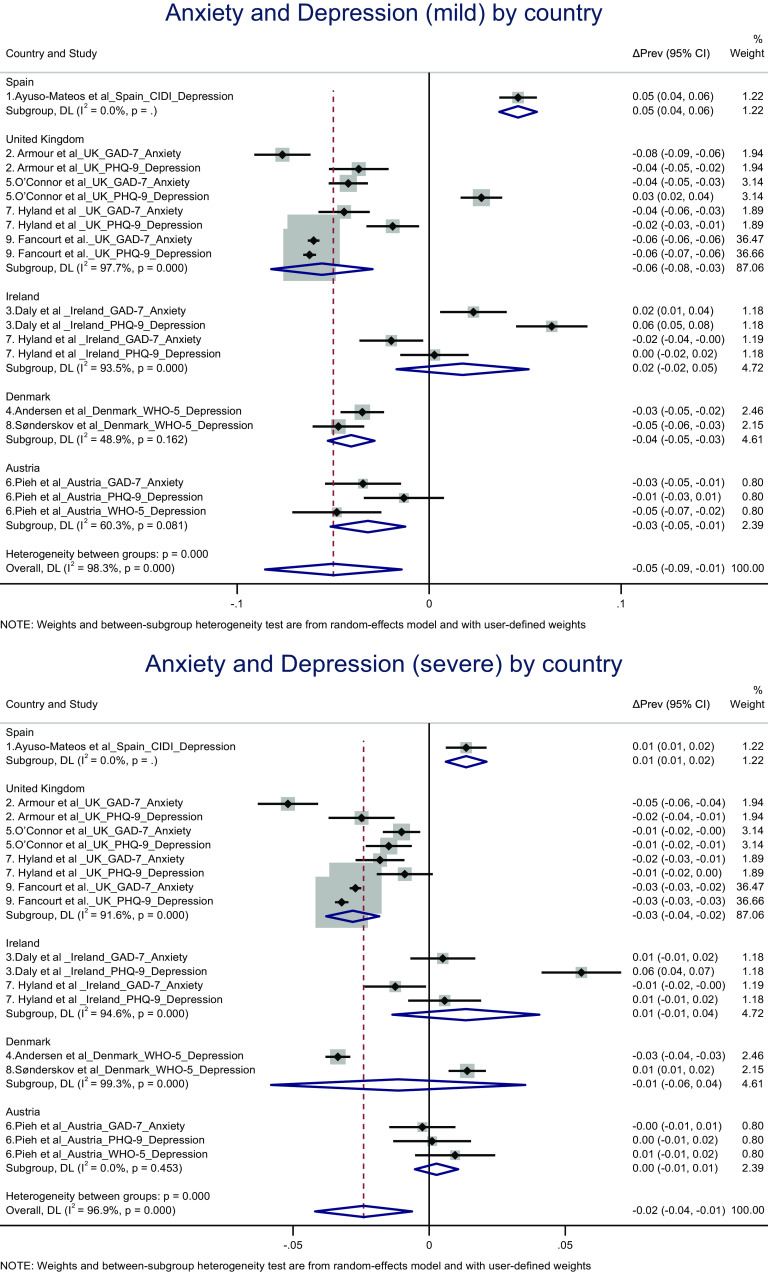
(A) Forest plot of random-effects meta-analysis results on early to during-pandemic changes in the prevalence of mild depression and anxiety, stratified by country. (B) Forest plot of random-effects meta-analysis results on early to during-pandemic changes in the prevalence of severe depression and anxiety, stratified by country. The results of individual studies and the pooled results are reported as the average early-to-during difference in the prevalence (ΔPrev) and its 95% confidence intervals (95% CI).

Among the studies with pre-pandemic data, country-specific analyses revealed no overall changes in the prevalence of mild or severe depression and anxiety combined. Among studies with early pandemic data, country-specific analyses revealed an overall decrease in mild depression and anxiety combined in the UK (ΔPrev = −0.056 [95% CI: −0.082, −0.029], *P* = 0.000; *I* ^2^ = 97.7%; and 95% prediction interval [−0.120, 0.008]) and Austria (ΔPrev = −0.056 [95% CI: −0.082, −0.029], *P* = 0.000; *I* ^2^ = 97.7%; and 95% prediction interval [−0.120, 0.008]) and an overall decrease in severe depression in the UK (ΔPrev = −0.028 [95% CI: −0.038, −0.018], *P* = 0.000; *I* ^2^ = 91.6%; and 95% prediction interval [−0.120, 0.008]) (see Figure 3A,B).

### Relationship between changes in the prevalence of mild and severe anxiety and depression with social distancing and school restrictions

The results in the following are reported as meta-regression coefficients for the change in the prevalence of anxiety and depression (coeff.), where the beta estimate denotes the effect 1-unit change in index has on the proportion of change in the outcome. Analyses are conducted for datasets with pre- and early pandemic data together. Through the meta-regression, we found that changes in school restrictions were significantly and positively associated with changes in the prevalence of mild anxiety and depression combined (coeff. = 9.41 × 10^−5^; *P* = 0.038). School restrictions and social distancing measures were not significantly associated with severe levels of anxiety and depression combined, or alone (Table 2). Additional adjustment for study quality did not alter the results (see Supplementary Table S11).

**Table 2.** Meta-regression coefficients for the change in the prevalence of anxiety and depression in relation to changes in social distancing and school restrictions for the COVID-19 pandemic

			Mild			
	Anxiety and depression combined		Anxiety		Depression	
	Coeff.	*P*-value	Coeff.	*P*-value	Coeff.	*P*-value
School restrictions	9.41 × 10^−5^	0.038*	4.80 × 10^−5^	0.545	9.54 × 10^−5^	0.117
Social distancing	5.09 × 10^−5^	0.144	−1.22 × 10^−5^	0.819	8.89 × 10^−5^	0.042

			Severe			
	Anxiety and depression combined		Anxiety		Depression	
	Coeff.	*P*-value	Coeff.	*P*-value	Coeff.	*P*-value
School restrictions	4.34 × 10^−5^	0.221	6.69 × 10^−5^	0.212	2.47 × 10^−5^	0.606
Social distancing	−2.21 × 10^−5^	0.424	3.71 × 10^−5^	0.382	−3.74 × 10^−5^	0.918

*Note*: For the analysis, data from both pre-to-during and early-to-during pandemic were used together.

## Discussion

This meta-analysis examined changes in mild and severe CMD prevalence rates in eight European countries from before/early pandemic to during the COVID-19 pandemic. The findings indicate no significant changes in the prevalence of mild/severe depression and/or anxiety when assessed from pre-pandemic to during the pandemic. However, among studies with data from early pandemic, significant decreases in the prevalence were found for mild anxiety and depression combined, mild anxiety alone, severe anxiety and depression combined, and severe anxiety alone. Similar results were also observed for specific age, sex, and national subgroups. Another finding was the positive association between school restrictions and *mild* depression and anxiety combined and between social distancing and *mild* depression, though with small effects.

Our findings diverge from previous studies of changes in CMD during the initial stages of the pandemic, which mainly suggested that there was an increase in the prevalence of major depressive disorder and generalized anxiety disorder [3, 12, 46, 47]. However, unlike previous studies that typically relied on single threshold values for depression and anxiety prevalence [24, 35, 36], the present study utilized two separate thresholds (i.e., mild and severe). Moreover, the longer follow-up periods of the included studies have allowed us to study additional nuances in these changes and associations.

Indeed, in our study mild anxiety and depression decreased across various demographic groups and most countries from early to during the pandemic. This aligns with findings from the UK, where clinically diagnosed anxiety and depression decreased over time as pandemic restrictions eased [30]. Several explanations could account for this. It is possible that many were not significantly impacted by the pandemic in the longer term [48] and the initial rise in anxiety and depression seen in the early pandemic literature reflects short-term changes. Another explanation may be that individuals developed resilience as the pandemic progressed and it became the “new normal” [49]. Additionally, our findings are paralleled by two other meta-analyses of studies with pre-pandemic to during pandemic data that found no changes in the prevalence of a broader range of mental disorders and symptoms after mid-2020, although there was an initial increase in the prevalence at the start of the pandemic [14, 15].

Our study indicated that increased school restrictions were associated with small increases in mild depression and anxiety combined, and increased social distancing was associated with small increases in mild depression. This is in line with a meta-analysis of 25 studies that associated lockdown measures with increases in depression and anxiety [49]. Also, studies have found increased distress levels in parents and children due to school closures [50, 51], possibly resulting from the challenges of balancing personal life, work, and children at home, which can hinder their ability to provide adequate support and contribute to psychological symptoms among children [50]. However, our findings contrast with a study across 33 countries, which found that the prevalence of depression was significantly lower in countries with promptly implemented stringent restrictions [52], but our study assessed prevalence at later time points, when restriction measures had already been implemented for an extended time.

### Strengths and limitations

This meta-analysis has allowed us to assess the effect of the COVID-19 pandemic on CMDs in the European population. Relatively homogenous healthcare systems with universal access across European countries enables valuable multinational comparisons, considering their varied containment strategies for COVID-19. Another strength of our study was that we collected raw or aggregated data from the study authors, which allowed for fewer restrictions to the data analysis in comparison to if we had only relied on data that were reported in the published studies.

There are several limitations to this study. Our objective to assess CMD at two levels required the use of raw/aggregated data. This took us long time to obtain, which in turn constrained our capacity to conduct a systematic literature search in several databases within a reasonable time frame. However, the limiting of our search allowed us to access and work with raw/aggregated data from the included studies, which is a strength that may in part outweigh the limitation of the search being non-systematic. The data are unweighted, so the results may not accurately represent the population being studied, and pre-/early and during demographics are thus not necessarily comparable. Moreover, we chose to define the pre-pandemic period as any time point up until March 2020. This definition of our pre-pandemic time point may impact comparability of our results with other studies using different time points. The choice of date for pre-pandemic periods in our study however will not affect the results regarding associations between lockdown measures and outcomes. Finally, it is noteworthy that all results presented large heterogeneity, which may be due to several possible confounding factors that vary between studies and settings, such as disparities of populations, heterogeneity of the outcomes measures used, and differences of the healthcare systems and policies implemented in the different countries. Therefore, the generalizability of the present findings is limited, and additional studies are needed to better understand how pandemic restrictions affect CMD.

## Conclusion

This study suggests that as the COVID-19 pandemic progressed in Europe, there was a decline in mild forms of emotional distress when compared to the initial increase in early pandemic, while rates of depression and anxiety remained stable. When comparing to pre-pandemic prevalence, however, there was no change. However, increased school restrictions and social distancing were found to be associated with small increases in mild anxiety and depression but not in severe levels. These findings support the importance of strong governance when implementing periodic and robust restriction measures to combat the spread of COVID-19.

### Registration and protocol

The study protocol has been registered and can be accessed on PROSPERO, with registration number CRD42022343130.

## Supporting information

Lok et al. supplementary material 1 Lok et al. supplementary material

Lok et al. supplementary material 2 Lok et al. supplementary material

## Supplementary material

The supplementary material for this article can be found at https://doi.org/10.1192/j.eurpsy.2023.2467.
