# Supplementary material for: Changes in anxiety and depression during the COVID-19 pandemic in the European population: A meta-analysis of changes and associations with restriction policies
Source: Eur Psychiatry. 2023 Oct 26;66(1):e87. doi: 10.1192/j.eurpsy.2023.2467 (PMC10755582; doi:10.1192/j.eurpsy.2023.2467)
Supplement: Lok et al. supplementary material 1 — Lok et al. supplementary material [file S0924933823024677sup001.docx]

**S1: Search Strings, Pub Med**

**PubMed search performed on April 1^st^ 2022:**

("Mental health"[Title/Abstract] OR "Mental disorders"[Title/Abstract] OR "Anxiety

Disorders"[MeSH Terms] OR "Depressive Disorder"[MeSH Major Topic] OR "anxiety"[Title/Abstract] OR "depress*"[Title/Abstract] OR "Mental disorder"[Title/Abstract] OR "psycholog*"[Title/Abstract]) AND ("novel coronavirus"[Title/Abstract] OR "COVID"[Title/Abstract] OR "covid-19"[Title/Abstract]

OR "covid19"[Title/Abstract] OR "covid19"[Title/Abstract] OR "nCoV"[Title/Abstract]

OR "novel CoV"[Title/Abstract] OR "2019nCoV"[Title/Abstract] OR

"Coronavirus"[Title/Abstract] OR "coronavi*"[Title/Abstract] OR "SARSCOV-

2"[Title/Abstract] OR "SARSCoV2"[Title/Abstract] OR "SARS CoV2"[Title/Abstract]

OR "outbreak"[Title/Abstract] OR "epidemic"[Title/Abstract] OR

"pandemic"[Title/Abstract] OR "Coronavirus"[MeSH Terms] OR "covid19"[Supplementary Concept] OR "Pandemics"[MeSH Terms] OR "severe acute respiratory syndrome coronavirus 2"[Supplementary Concept] OR "covid-

19"[Supplementary Concept]) AND ("prevalen*"[Title/Abstract] OR

"Prevalence"[MeSH Terms] OR "impact"[Title/Abstract] OR "effect"[Title/Abstract] OR

"outcome"[Title/Abstract] OR "percentage"[Title/Abstract]) AND ("Russia*"[MeSH

Terms] OR "Germany*"[MeSH Terms] OR "United Kingdom"[MeSH Terms] OR

"France"[MeSH Terms]) OR "Italy"[MeSH Terms] OR "Spain"[MeSH Terms] OR

"Ukraine"[MeSH Terms] OR "Poland"[MeSH Terms] OR "Romania"[MeSH Terms] OR "Netherlands"[MeSH Terms] OR "Belgium"[MeSH Terms] OR "Czech

Republic"[MeSH Terms] OR "Greece"[MeSH Terms] OR "Portugal"[MeSH Terms]

OR "Sweden"[MeSH Terms] OR "Hungary"[MeSH Terms] OR "Belarus"[MeSH

Terms] OR "Austria"[MeSH Terms] OR "Serbia"[MeSH Terms] OR

"Switzerland"[MeSH Terms] OR "Bulgaria"[MeSH Terms] OR "Denmark"[MeSH

Terms] OR "Finland"[MeSH Terms] OR "Slovakia"[MeSH Terms] OR

"Norway"[MeSH Terms] OR "Ireland"[MeSH Terms] OR "Croatia"[MeSH Terms] OR

"Moldova"[MeSH Terms] OR "Bosnia and Herzegovina" [MeSH Terms] OR

"Albania"[MeSH Terms] OR "Lithuania"[MeSH Terms] OR " Republic of North

Macedonia "[MeSH Terms] OR "Slovenia"[MeSH Terms] OR "Latvia"[MeSH Terms]

OR "Estonia"[MeSH Terms] OR "Montenegro"[MeSH Terms] OR

"Luxembourg"[MeSH Terms] OR "Malta"[MeSH Terms] OR "Iceland"[MeSH Terms]

OR "Andorra"[MeSH Terms] OR "Monaco"[MeSH Terms] OR "Liechtenstein"[MeSH

Terms] OR "San Marino"[MeSH Terms] OR " Vatican City "[MeSH Terms] OR "

Europe "[MeSH Terms] OR " Scandinavian and Nordic Countries "[MeSH Terms] OR " Europe, Eastern "[MeSH Terms] OR " Europe, Eastern "[MeSH Terms] OR " Baltic States "[MeSH Terms OR " Mediterranean Islands "[MeSH Terms) AND ("CES-D"[Title/Abstract] OR "Center for Epidemiologic Studies Depression

Scale"[Title/Abstract] OR "CIDI"[Title/Abstract] OR "Composite International

Diagnostic Interview"[Title/Abstract] OR "DASS-A"[Title/Abstract] OR "Depression

Anxiety Stress Scales-anxiety"[Title/Abstract] OR "DASS-D"[Title/Abstract] OR

"Depression Anxiety Stress Scales-depression"[Title/Abstract] OR "GAD7"[Title/Abstract] OR "General Anxiety Disorder-7"[Title/Abstract] OR "GAD9"[Title/Abstract] OR "General Anxiety Disorder-9"[Title/Abstract] OR "GHQ12"[Title/Abstract] OR "12-Item General Health Questionnaire"[Title/Abstract] OR "HAD"[Title/Abstract] OR "hospital anxiety depression scale"[Title/Abstract] OR "HADS-Anxiety"[Title/Abstract] OR "hospital anxiety and depression scaleanxiety"[Title/Abstract] OR "HADS-depression"[Title/Abstract] OR "hospital anxiety and depression scale-depression"[Title/Abstract] OR "HBQ"[Title/Abstract] OR "MacArthur Health and Behavior Questionnaire"[Title/Abstract] OR

"K10"[Title/Abstract] OR "K6"[Title/Abstract] OR "Kessler Psychological Distress

Scale"[Title/Abstract] OR "MFQ"[Title/Abstract] OR "Mood Feelings Questionnaire"[Title/Abstract] OR "MHI-5"[Title/Abstract] OR "mental health inventory"[Title/Abstract] OR "MINI"[Title/Abstract] OR "Mini-International Neuropsychiatric Interview"[Title/Abstract] OR "PHQ-8"[Title/Abstract] OR "eight-item Patient Health Questionnaire depression scale"[Title/Abstract] OR "PHQ9"[Title/Abstract] OR "night item Patient Health Questionnaire depression scale"[Title/Abstract] OR "SCARED"[Title/Abstract] OR "Screen Child Anxiety Related Disorders"[Title/Abstract] OR "SMFQ"[Title/Abstract] OR "Short Mood

Feelings Questionnaire"[Title/Abstract] OR "WHO-5"[Title/Abstract] OR "World Health Organisation- Five Well-Being Index"[Title/Abstract])

**Filters:**

Humans, from 2021/1/30 - 3000/12/12

**S2: Outcome measures included and scale-specific thresholds used in the study:**

The outcome measure was changes in the prevalence of self-reported mild and severe anxiety and depression from before to during the COVID-19 pandemic. Mild and severe anxiety and depression were measured using the following standardized and validated measures: CIDI (Composite International Diagnostic Interview)(1); GAD-7 (Generalized Anxiety Disorder 7 items)(2); HADS-Anxiety (Hospital Anxiety and Depression Scale – Anxiety)(3); HADS-depression (Hospital Anxiety and Depression Scale – Depression)(3); MHI-5 (Mental Health Inventory 5)(4); PHQ-9 (Patient Health Questionnaire 9 items)(5); WHO-5 (World Health Organization 5 Well-Being Index)(6), and the cut-offs and recall periods for each scale are shown in the table below.

| **Assesment scale** | **Threshold for milder symtoms** | **Threshold for severe symtoms** | **Recall period** |
| --- | --- | --- | --- |
| CIDI | YES /NO | | 1 month |
| GAD-7 | ≥10 | ≥15 | 2 weeks |
| HADS | ≥8 | ≥11 | 1 week |
| MHI-5 | ≤72 | ≤60 | 4 weeks |
| PHQ-9 | ≥10 | ≥15 | 2 weeks |
| WHO-5 | ≤50 | ≤25 | 2 weeks |

**S3: Information about the corona containment index:**

The corona containment index was based on the Bayesian time-varying measurement model, which combined the CoronaNet COVID-19 Government Response Event Dataset and the Oxford COVID-19 Government Response Tracker to measure the intensity of government responses to COVID-19 across 6 distinct policy areas (7). The policy areas included the following: 1) general social distancing, capturing restrictions on individual mobility; 2) masks, capturing the wearing of face masks; 3) business restrictions, capturing restrictions on private commerce and industry; 4) schools restrictions, capturing restriction on education provision; 5) health monitoring, capturing monitoring of individual health statuses; and 6) health resources, capturing medical resources employed to treat the disease. Each index is given a value between 0 and 100, indicating strictness of that particular policy area.

**Table S4: Quality assessment with Newcastle-Ottawa Scale**

| **Database #** | **First author** | **NOS Q1** | **NOS Q2** | **NOS Q3** | **NOS Q4** | **NOS Q5** | **NOS Q6** | **NOS Q7** | **NOS Q8** | **Study quality** |
| --- | --- | --- | --- | --- | --- | --- | --- | --- | --- | --- |
| 1 | Ayuso-Mateos | 1 | 1 | 0 | 1 | 1 | 1 | 1 | 1 | 7 |
| 2 | Armour | 1 | 1 | 0 | 1 | 1 | 0 | 1 | 1 | 6 |
| 3 | Daly | 1 | 1 | 0 | 1 | 1 | 0 | 1 | 1 | 6 |
| 4 | Andersen | 1 | 1 | 0 | 1 | 1 | 0 | 1 | 1 | 6 |
| 5 | O’Connor | 1 | 1 | 0 | 1 | 1 | 0 | 1 | 1 | 6 |
| 6 | Pieh | 1 | 1 | 0 | 1 | 1 | 0 | 1 | 1 | 6 |
| 7 | Hyland | 1 | 1 | 0 | 1 | 1 | 1 | 1 | 1 | 7 |
| 8 | Sonderskov | 1 | 1 | 0 | 1 | 1 | 0 | 1 | 1 | 6 |
| 9 | Fancourt | 1 | 1 | 0 | 1 | 1 | 0 | 1 | 1 | 6 |
| 10 | Knudsen | 1 | 1 | 0 | 1 | 1 | 1 | 1 | 1 | 7 |
| 11 | van der Velden | 1 | 1 | 0 | 1 | 1 | 0 | 1 | 1 | 6 |
| 12 | Widnall | 1 | 1 | 0 | 1 | 1 | 0 | 1 | 1 | 6 |
| 13 | Winkler | 1 | 1 | 0 | 1 | 1 | 0 | 1 | 1 | 6 |
| 14 | UKHLS | 1 | 1 | 0 | 1 | 1 | 1 | 1 | 1 | 7 |
| 15 | Briggs | 1 | 1 | 0 | 1 | 1 | 0 | 1 | 1 | 6 |

**Table S5** Random effects meta-analysis results on pre- to during-pandemic changes in prevalence of self-reported mild and severe depression and anxiety

|  | | |  | Mild |  |  |  |  |
| --- | --- | --- | --- | --- | --- | --- | --- | --- |
| Anxiety and Depression combined | | |  | Anxiety |  |  | Depression |  |
| effect ΔPrev (95% CI) / [predicted interval] | p-value | I^2^(%) | effect ΔPrev (95% CI) / [predicted interval] | p-value | I^2^(%) | effect ΔPrev (95% CI) / [predicted interval] | p-value | I^2^(%) |
| **-0.010 (-0.109, 0.088) / [-0.284, 0.264]** | **0.839** | **99.6%** | **-** | **-** | **-** | **-0.008 (-0.154, 0.139) / NA** | **0.919** | **99.8%** |
|  | | |  | Severe |  |  |  |  |
| Anxiety and Depression combined | | |  | Anxiety |  |  | Depression |  |
| effect ΔPrev (95% CI) / [predicted interval] | p-value | I^2^(%) | effect ΔPrev (95% CI) / [predicted interval] | p-value | I^2^(%) | effect ΔPrev (95% CI) / [predicted interval] | p-value | I^2^(%) |
| **0.002 (-0.046, 0.051) / [-0.109, 0.113]** | **0.923** | **99.2%** | **-0.005 (-0.085, 0.075) / NA** | **0.899** | **99.1%** | **0.006 (-0.080, 0.091) / NA** | **0.892** | **99.5%** |

**Note:** Boxes with ”-” indicate that not enough studies could be included in the meta-analysis for that variable. NA denotes pooled effects not applicable for estimating 95% prediction interval since the number of included studies is less than 5.

**Table S6** Random effects meta-analysis results on early to during-pandemic changes in prevalence of self-reported mild and severe depression and anxiety

|  | | |  | Mild |  |  |  |  |
| --- | --- | --- | --- | --- | --- | --- | --- | --- |
| Anxiety and Depression combined | | |  | Anxiety |  |  | Depression |  |
| effect ΔPrev (95% CI) / [predicted interval] | p-value | I^2^(%) | effect ΔPrev (95% CI) / [predicted interval] | p-value | I^2^(%) | effect ΔPrev (95% CI) / [predicted interval] | p-value | I^2^(%) |
| **-0.050 (-0.085, -0.014) / [-0.133, 0.033]** | **0.006*** | **98.3%** | **-0.055 (-0.094, -0.017) / [-0.137, 0.026]** | **0.005*** | **95.3%** | **-0.045 (-0.112, 0.022) / [-0.181, 0.091]** | **0.187** | **98.7%** |
|  | | |  | Severe |  |  |  |  |
| Anxiety and Depression combined | | |  | Anxiety |  |  | Depression |  |
| effect ΔPrev (95% CI) / [predicted interval] | p-value | I^2^(%) | effect ΔPrev (95% CI) / [predicted interval] | p-value | I^2^(%) | effect ΔPrev (95% CI) / [predicted interval] | p-value | I^2^(%) |
| **-0.024 (-0.042, -0.006) / [-0.066, 0.017]** | **0.008*** | **96.9%** | **-0.025** **(-0.047, -0.003) /** **[-0.071, 0.021]** | **0.024*** | **93.3%** | **-0.023 (-0.056, 0.009) / [-0.089, 0.042]** | **0.160** | **97.8%** |

**Note:** Boxes with ”-” indicate that not enough studies could be included in the meta-analysis for that variable. * Denotes significant results.

**Table S7** Random effects meta-analysis results on pre- to during-pandemic changes in prevalence of self-reported mild depression and anxiety stratified by demographic characteristics

|  | | **Mild** | | | | | | | |  |
| --- | --- | --- | --- | --- | --- | --- | --- | --- | --- | --- |
|  |  | Anxiety and Depression combined | | | Anxiety | | | Depression | | |
|  |  | effect ΔPrev (95% CI) / [predicted interval] | p-value | I^2^(%) | effect ΔPrev (95% CI) / [predicted interval] | p-value | I^2^(%) | effect ΔPrev (95% CI) / [predicted interval] | p-value | I^2^(%) |
| **By age** | |  | | |  |  |  |  |  |  |
| 0-18 | | **-0.013 (-0.074, 0.047) / NA** | **0.669** | **95.3%** | - | - | - | - | - | - |
| 19-64 | | **-0.021 (-0.118, 0.076) / NA** | **0.672** | **99.4%** | - | - | - | - | - | - |
| 65+ | | **0.017 (-0.081, 0.115) / NA** | **0.732** | **99.6%** | - | - | - | - | - | - |
| **By sex** | |  | | |  |  |  |  |  |  |
| Male | | **-0.013 (-0.090, 0.065) / [-0.230, 0.205]** | **0.746** | **98.9%** | - | - | - | **-0.013 (-0.131, 0.104) / NA** | **0.822** | **99.5%** |
| Female | | **-0.012 (-0.134, 0.109) / [-0.347, 0.322]** | **0.842** | **99.5%** | - | - | - | **-0.008 (-0.185, 0.169) / NA** | **0.930** | **99.7%** |
| **By sex and age** | |  | | |  |  |  |  |  |  |
| Male | 0-18 | **-0.037 (-0.081, 0.007) / NA** | **0.099** | **83.8%** | - | - | - | **-0.028 (-0.093, 0.037) / NA** | **0.399** | **89.5%** |
|  | 19-64 | - | - | - | - | - | - | - | - | - |
|  | 65+ | - | - | - | - | - | - | - | - | - |
| Female | 0-18 | **-0.013 (-0.090, 0.064) / NA** | **0.736** | **94.6%** | - | - | - | - | - | - |
|  | 19-64 | - | - | - | - | - | - | - | - | - |
|  | 65+ | - | - | - | - | - | - | - | - | - |

**Note:** Boxes with ”-” indicate that not enough studies could be included in the meta-analysis for that variable. NA denotes pooled effects not applicable for estimating 95% prediction interval since the number of included studies is less than 5.

**Table S8** Random effects meta-analysis results on pre- to during-pandemic changes in prevalence of self-reported severe depression and anxiety stratified by demographic characteristics

|  | | **Severe** | | | | | | | |  |
| --- | --- | --- | --- | --- | --- | --- | --- | --- | --- | --- |
|  |  | Anxiety and Depression combined | | | Anxiety | | | Depression | | |
|  |  | effect ΔPrev (95% CI) / [predicted interval] | p-value | I^2^(%) | effect ΔPrev (95% CI) / [predicted interval] | p-value | I^2^(%) | effect ΔPrev (95% CI) / [predicted interval] | p-value | I^2^(%) |
| **By age** | |  | | |  |  |  |  |  |  |
| 0-18 | | **-0.015 (-0.053, 0.024) / NA** | **0.463** | **93.0%** | - | - | - | - | - | - |
| 19-64 | | **0.008 (-0.062, 0.077) / [-0.168, 0.183]** | **0.828** | **99.5%** | - | - | - | **0.009 (-0.112, 0.130) / NA** | **0.884** | **99.7%** |
| 65+ | | **0.009 (-0.027, 0.044) / NA** | **0.631** | **98.5%** | - | - | - | - | - | - |
| **By sex** | |  | | |  |  |  |  |  |  |
| Male | | **0.003 (-0.040, 0.047) / [-0.098, 0.105]** | **0.884** | **98.5%** | **0.006 (-0.060, 0.071) / NA** | **0.869** | **98.0%** | **0.005 (-0.073, 0.083)** | **0.905** | **99.1%** |
| Female | | **-0.002 (-0.046, 0.043) / [-0.103, 0.100]** | **0.938** | **98.0%** | **-0.008 (-0.063, 0.047) / NA** | **0.778** | **96.5%** | **0.001 (-0.079, 0.081) / NA** | **0.977** | **98.9%** |
| **By sex and age** | |  | | |  |  |  |  |  |  |
| Male | 0-18 | **-0.024 (-0.058, 0.009) / NA** | **0.158** | **86.6%** | - | - | - | - | - | - |
|  | 19-64 | **0.008 (-0.049, 0.064) / [-0.139, 0.155]** | **0.790** | **99.0%** | - | - | - | **0.007 (-0.094, 0.107) / NA** | **0.892** | **99.4%** |
|  | 65+ | **0.006 (-0.019, 0.031) / NA** | **0.635** | **93.5%** | - | - | - | - | - | - |
| Female | 0-18 | **-0.019 (-0.054, 0.017) / NA** | **0.303** | **83.6%** | - | - | - | - | - | - |
|  | 19-64 | **0.002 (-0.070, 0.073) / [-0.176, 0.179]** | **0.965** | **99.0%** | - | - | - | **0.001 (-0.121, 0.124) / NA** | **0.982** | **99.5%** |
|  | 65+ | **0.003 (-0.031, 0.036) / NA** | **0.881** | **95.0%** | - | - | - | - | - | - |

**Note:** Boxes with ”-” indicate that not enough studies could be included in the meta-analysis for that variable. NA denotes pooled effects not applicable for estimating 95% prediction interval since the number of included studies is less than 5.

**Table S9** Random effects meta-analysis results on early to during-pandemic changes in prevalence of self-reported mild depression and anxiety stratified by demographic characteristics

|  | |  | | | |  | **Mild** |  |  |  |  |
| --- | --- | --- | --- | --- | --- | --- | --- | --- | --- | --- | --- |
|  |  | Anxiety and Depression combined | | | | Anxiety | | | Depression | | |
|  |  | Effect ΔPrev (95% CI) / [predicted interval] | p-value | | I^2^(%) | effect ΔPrev (95% CI) / [predicted interval] | p-value | I^2^(%) | effect ΔPrev (95% CI) / [predicted interval] | p-value | I^2^(%) |
| **By age** | |  | | | |  |  |  |  |  |  |
| 0-18 | | **-0.119 (-0.380.0.141) / [-1.112, 0.874]** | **0.368** | | **95.6%** | - | - | - | **-0.052 (-0.443, 0.339) / NA** | **0.794** | **96.8%** |
| 19-64 | | **-0.059 (-0.106, -0.013) / [-0.171, 0.052]** | **0.012*** | | **98.6%** | **-0.067 (-0.121, -0.013) / [-0.185, 0.050]** | **0.015*** | **97.0%** | **-0.053 (-0.138, 0.033) / [-0.229, 0.123]** | **0.262** | **99.0%** |
| 65+ | | **-0.016 (-0.032, 0.000) / [-0.051, 0.019]** | **0.056** | | **82.4%** | **-0.017 (-0.048, 0.014) / [-0.079, 0.045]** | **0.288** | **82.8%** | **-0.016 (-0.043, 0.012) / [-0.067, 0.037]** | **0.305** | **83.9%** |
| **By sex** | |  | | | |  |  |  |  |  |  |
| Male | | **-0.039 (****-0.074, -0.004) / [-0.119, 0.041]** | **0.029*** | | **96.7%** | **-0.039 (-0.073, -0.004) / [-0.111, 0.034]** | **0.029*** | **89.9%** | **-0.038 (-0.111, 0.033) / [-0.182, 0.104]** | **0.351** | **98.0%** |
| Female | | **-0.059** **(-0.099, -0.019) / [-0.154, 0.035]** | **0.004*** | | **97.3%** | **-0.070 (-0.112, -0.028) / [-0.160, 0.020]** | **0.001*** | **92.4%** | **-0.052 (-0.120, 0.20) / [-0.193, 0.093]** | **0.182** | **97.9%** |
| **By sex and age** | |  | | | |  |  |  |  |  |  |
| Male | 0-18 | **-0.052 (-0.351, 0.247) / [-1.152, 1.048]** | | **0.734** | **91.3%** | - | - | - | **-0.032 (-0.411, 0.347) / NA** | **0.868** | **90.6%** |
|  | 19-64 | **-0.052 (-0.103, -0.001) / [-0.173, 0.069]** | | **0.046*** | **97.6%** | **-0.054 (-0.115, 0.006) / [-0.184, 0.075]** | **0.077** | **95.2%** | **-0.048 (-0.148, 0.048) / [-0.250, 0.150]** | **0.364** | **98.4%** |
|  | 65+ | **-0.006 (-0.022, 0.010) / [-0.040, 0.028]** | | **0.461** | **75.1%** | **-0.003 (-0.033, 0.028) / [-0.062, 0.056]** | **0.838** | **75.3%** | **-0.008 (-0.028, 0.021) / [-0.064, 0.047]** | **0.642** | **78.2%** |
| Female | 0-18 | **-0.153 (-0.406, 0.100) / [-1.117, 0.811]** | | **0.237** | **93.3%** | - | - | - | **-0.068 (-0.457, 0.322) / NA** | **0.733** | **95.4%** |
|  | 19-64 | **-0.066 (-0.113, -0.019) / [-0.179, 0.048]** | | **0.006*** | **97.4%** | **-0.078 (-0.130, -0.026) / [-0.192, 0.036]** | **0.003*** | **94.2%** | **-0.057 (-0.137, 0.027) / [-0.226, 0.115]** | **0.205** | **98.1%** |
|  | 65+ | **-0.037 (-0.069, -0.005) / [-0.111, 0.036]** | | **0.023*** | **89.6%** | **-0.036 (-0.074, 0.002) / [-0.111, 0.039]** | **0.062** | **72.1%** | **-0.043 (-0.092, 0.016) / [-0.152, 0.076]** | **0.126** | **91.9%** |

**Note:** Boxes with ”-” indicate that not enough studies could be included in the meta-analysis for that variable. NA denotes pooled effects not applicable for estimating 95% prediction interval since the number of included studies is less than 5. * Denotes significant results.

**Table S10** Random effects meta-analysis results on early to during-pandemic changes in prevalence of self-reported severe depression and anxiety stratified by demographic characteristics

|  | | **Severe** | | | | | | | | | | | | | | | |  |
| --- | --- | --- | --- | --- | --- | --- | --- | --- | --- | --- | --- | --- | --- | --- | --- | --- | --- | --- |
|  |  | Anxiety and Depression combined | | | | Anxiety | | | | | | Depression | | | | | |  |
|  |  | effect ΔPrev (95% CI) / [predicted interval] | p-value | I^2^(%) | | effect ΔPrev (95% CI) / [predicted interval] | | p-value | | I^2^(%) | | effect ΔPrev (95% CI) / [predicted interval] | | p-value | | I^2^(%) | |  |
| **By age** | |  | | | |  | |  | |  | |  | |  | |  | |  |
| 0-18 | | **-0.116 (-0.232, -0.001) / [-0.540, 0.308]** | **0.048*** | **90.4%** | | - | | - | | - | | **-0.131 (-0.270, 0.09) / NA** | | **0.066** | | **90.1%** | |  |
| 19-64 | | **-0.031 (****-0.057, -0.005) / [-0.094, 0.032]** | **0.020*** | **97.8%** | | **-0.032 (-0.062, -0.001) / [-0.097, 0.034]** | | **0.042*** | | **95.6%** | | **-0.030 (-0.079, 0.017) / [-0.130, 0.068]** | | **0.278** | | **98.6%** | |  |
| 65+ | | **-0.002 (-0.011, 0.008) / [-0.022, 0.019]** | **0.722** | **82.5%** | | **-0.004 (-0.022, 0.013) / [-0.039, 0.030]** | | **0.633** | | **80.6%** | | **0.002 (-0.016, 0.016) / [-0.030, 0.030]** | | **0.839** | | **74.6%** | |  |
| **By sex** | |  | | | |  | |  | |  | |  | |  | |  | |  |
| Male | | **-0.016 (-0.032, 0.001) / [-0.053, 0.022]** | **0.059** | **94.0%** | | **-0.014 (-0.034, 0.005) / [-0.055, 0.026]** | | **0.152** | | **87.5%** | | **-0.017 (-0.048, 0.014) / [-0.078, 0.044]** | | **0.381** | | **96.2%** | |  |
| Female | | **-0.031 (****-0.051, -0.012) /** **[-0.078, 0.015]** | **0.002*** | **94.6%** | | **-0.034 (-0.059, -0.010) / [-0.087, 0.018]** | | **0.007*** | | **89.3%** | | **-0.028 (-0.063, 0.006) / [-0.099, 0.042]** | | **0.155** | | **95.7%** | |  |
| **By sex and age** | |  | | | |  | |  | |  | |  | |  | |  | |  |
| Male | 0-18 | **-0.032 (-0.216, 0.152) / [-0.700, 0.636]** | **0.732** | | **91.3%** | | - | | - | | - | | **-0.034 (-0.266, 0.199) / NA** | | **0.777** | | **91.3%** | |
|  | 19-64 | **-0.024 (-0.051, 0.002) / [-0.087, 0.038]** | **0.072** | | **96.1%** | | **-0.021 (-0.053, 0.010) / [-0.088, 0.046]** | | **0.180** | | **92.9%** | | **-0.027 (-0.075, 0.021) / [-0.125, 0.071]** | | **0.357** | | **97.5%** | |
|  | 65+ | **0.003 (-0.004, 0.010) / [-0.011, 0.018]** | **0.365** | | **62.1%** | | **0.001 (-0.013, 0.015) / [-0.028, 0.030]** | | **0.895** | | **66.9%** | | **0.005 (-0.005, 0.016) / [-0.014, 0.025]** | | **0.455** | | **60.0%** | |
| Female | 0-18 | **-0.153 (-0.254, -0.052) / [-0.506, 0.200]** | **0.003*** | | **82.1%** | | - | | - | | - | | **-0.173 (-0.325, -0.021) / NA** | | **0.026*** | | **88.4%** | |
|  | 19-64 | **-0.037 (-0.063, -0.010) / [-0.101, 0.028]** | **0.007*** | | **95.9%** | | **-0.040 (-0.072, -0.008) / [-0.109, 0.029]** | | **0.014*** | | **92.0%** | | **-0.033 (-0.081, 0.014) / [-0.133, 0.065]** | | **0.218** | | **97.0%** | |
|  | 65+ | **-0.011 (-0.025, 0.003) / [-0.044, 0.022]** | **0.137** | | **83.3%** | | **-0.011 (-0.034, 0.011) / [-0.056, 0.033]** | | **0.330** | | **72.5%** | | **-0.009 (-0.033, 0.012) / [-0.058, 0.037]** | | **0.430** | | **82.4%** | |

**Note:** Boxes with ”-” indicate that not enough studies could be included in the meta-analysis for that variable. NA denotes pooled effects not applicable for estimating 95% prediction interval since the number of included studies is less than 5. * Denotes significant results.

**Table S11.** Meta-regression coefficients for the change in prevalence of anxiety and depression in relation to changes in social distancing and school restrictions for the COVID-19 pandemic with additional adjustment for study quality.

|  |  | |  | Mild | |  | | |
| --- | --- | --- | --- | --- | --- | --- | --- | --- |
|  | Anxiety and Depression combined | | | Anxiety | | | Depression | |
|  | Coeff. | p-value | | Coeff. | p-value | | Coeff. | p-value |
| School restrictions | 1.10 x 10^-4^ | 0.040* | | 7.06 x 10^-5^ | 0.590 | | 1.04 x 10^-4^ | 0.126 |
| Social distancing | 6.52 x 10^-5^ | 0.129 | | -3.40 x 10^-5^ | 0.654 | | 1.14 x 10^-4^ | 0.028* |
|  |  | |  | Severe | |  | | |
|  | Anxiety and Depression combined | | | Anxiety | | | Depression | |
|  | Coeff. | p-value | | Coeff. | p-value | | Coeff. | p-value |
| School restrictions | 4.74 x 10^-5^ | 0.211 | | 8.87 x 10^-5^ | 0.114 | | 2.03 x 10^-5^ | 0.699 |
| Social distancing | -3.29 x 10^-5^ | 0.348 | | -0.0002973 | 0.578 | | -2.12 x 10^-5^ | 0.655 |

**Note**: For the analysis, data from both pre-to-during and early-to-during pandemic were used together.

* Denotes significant results.

## **Figure S1A:** Time plot graph of prevalence of mild anxiety and depression combined from studies with pre-pandemic data


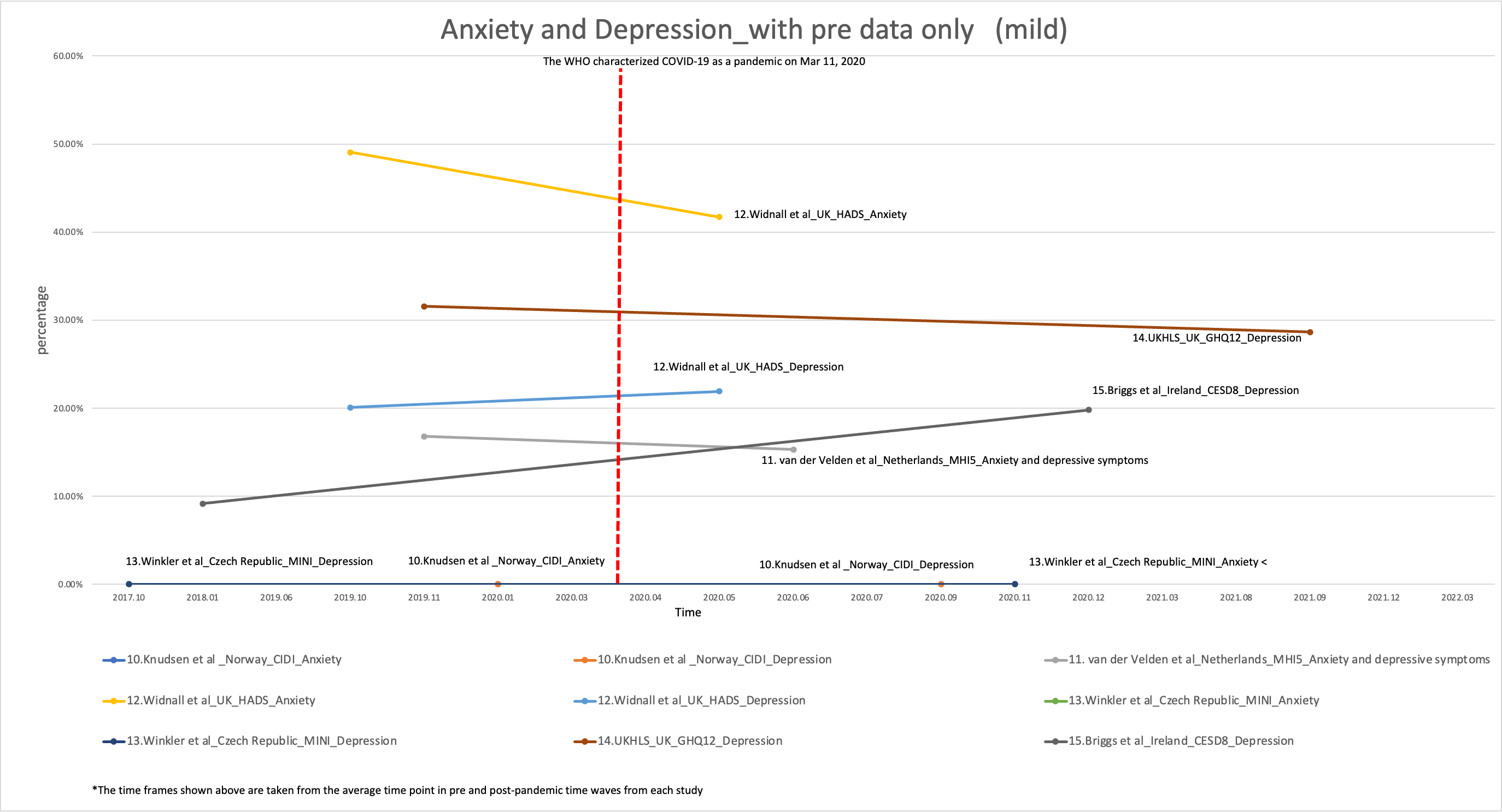


## **Figure S1B:** Time plot graph of prevalence of severe anxiety and depression combined from studies with pre-pandemic data


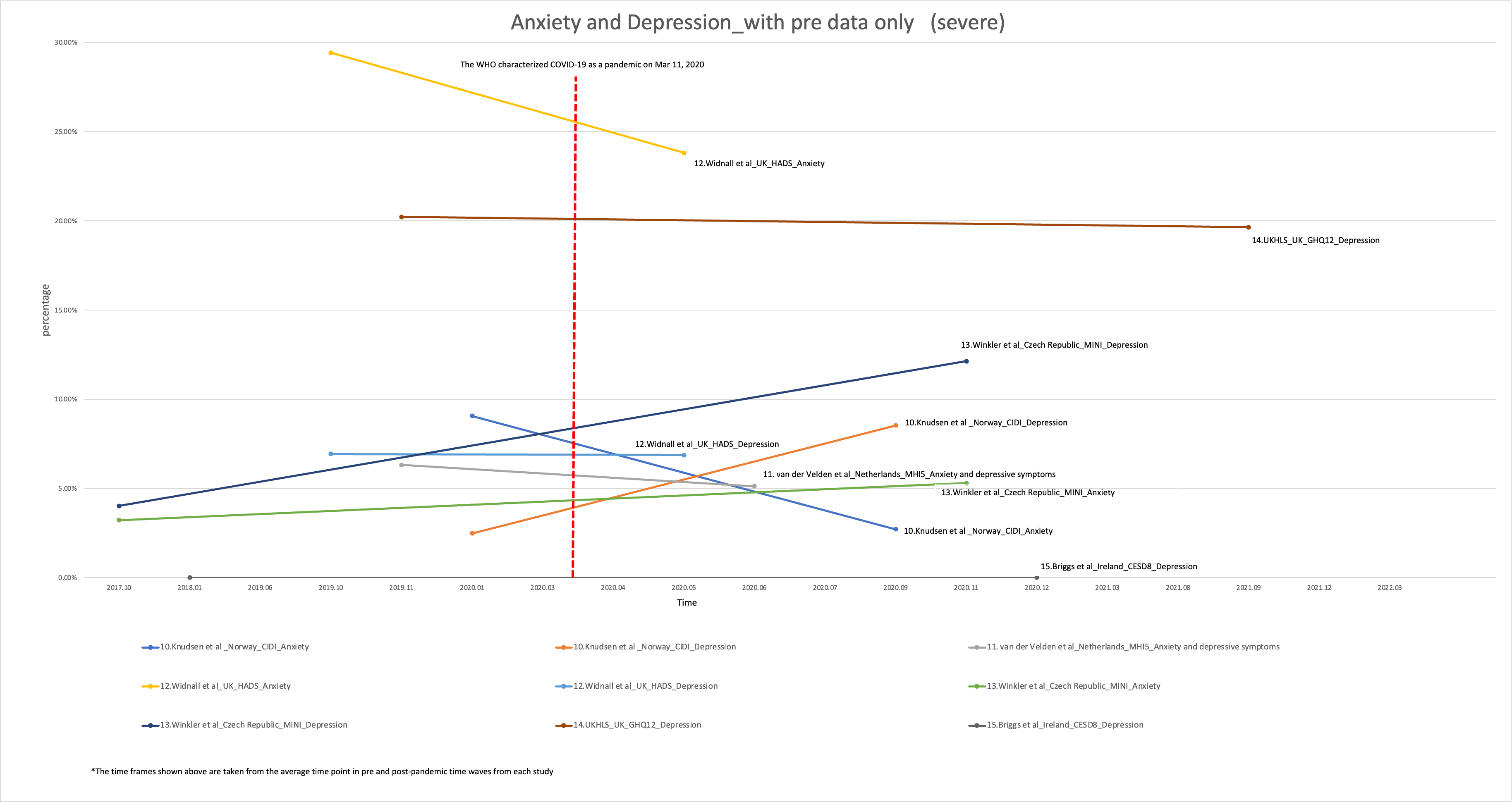


## **Figure S1C:** Time plot graph of prevalence of mild anxiety from studies with pre-pandemic data


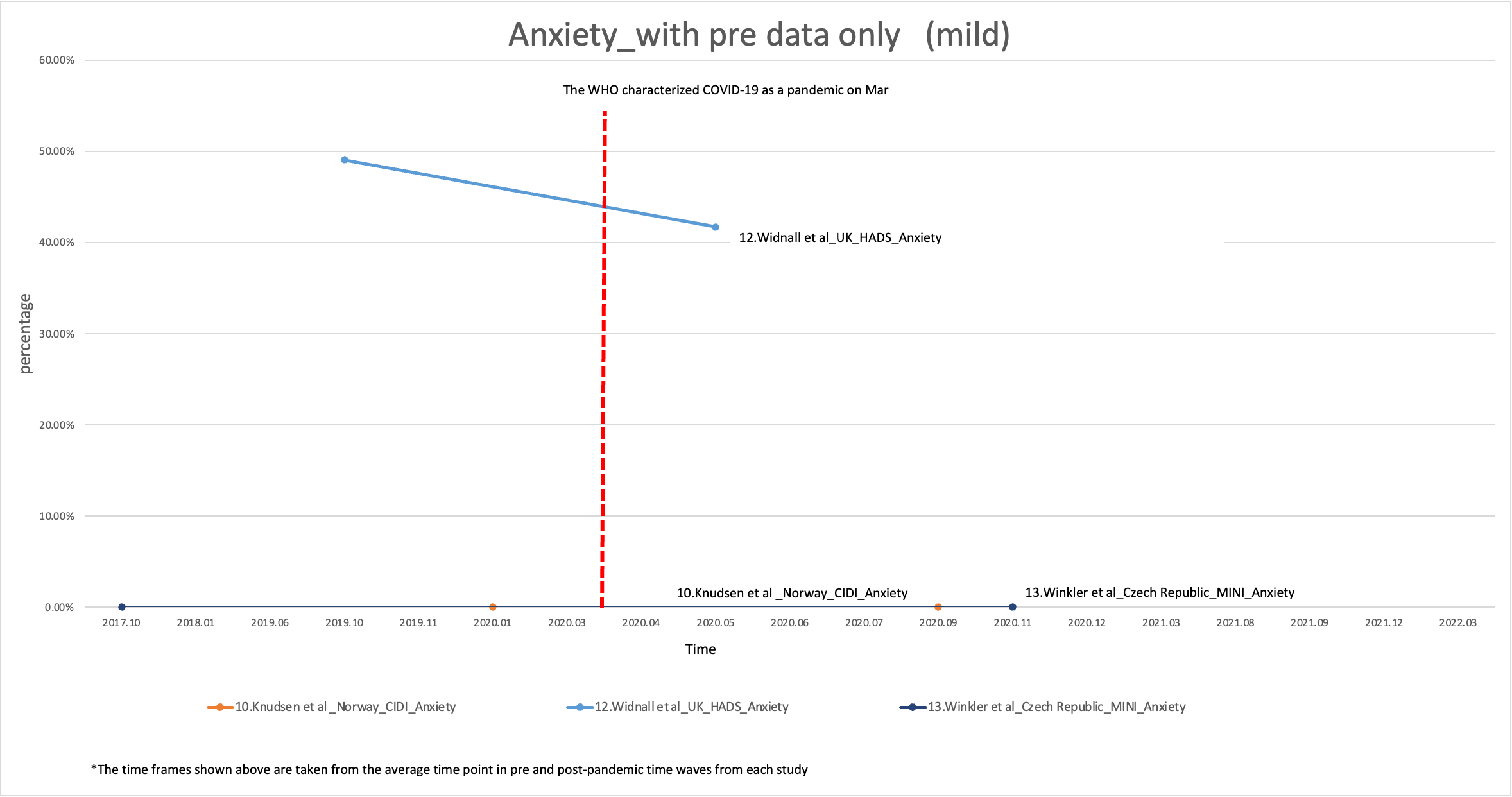


**Figure S1D:** Time plot graph of prevalence of severe anxiety from studies with pre-pandemic data


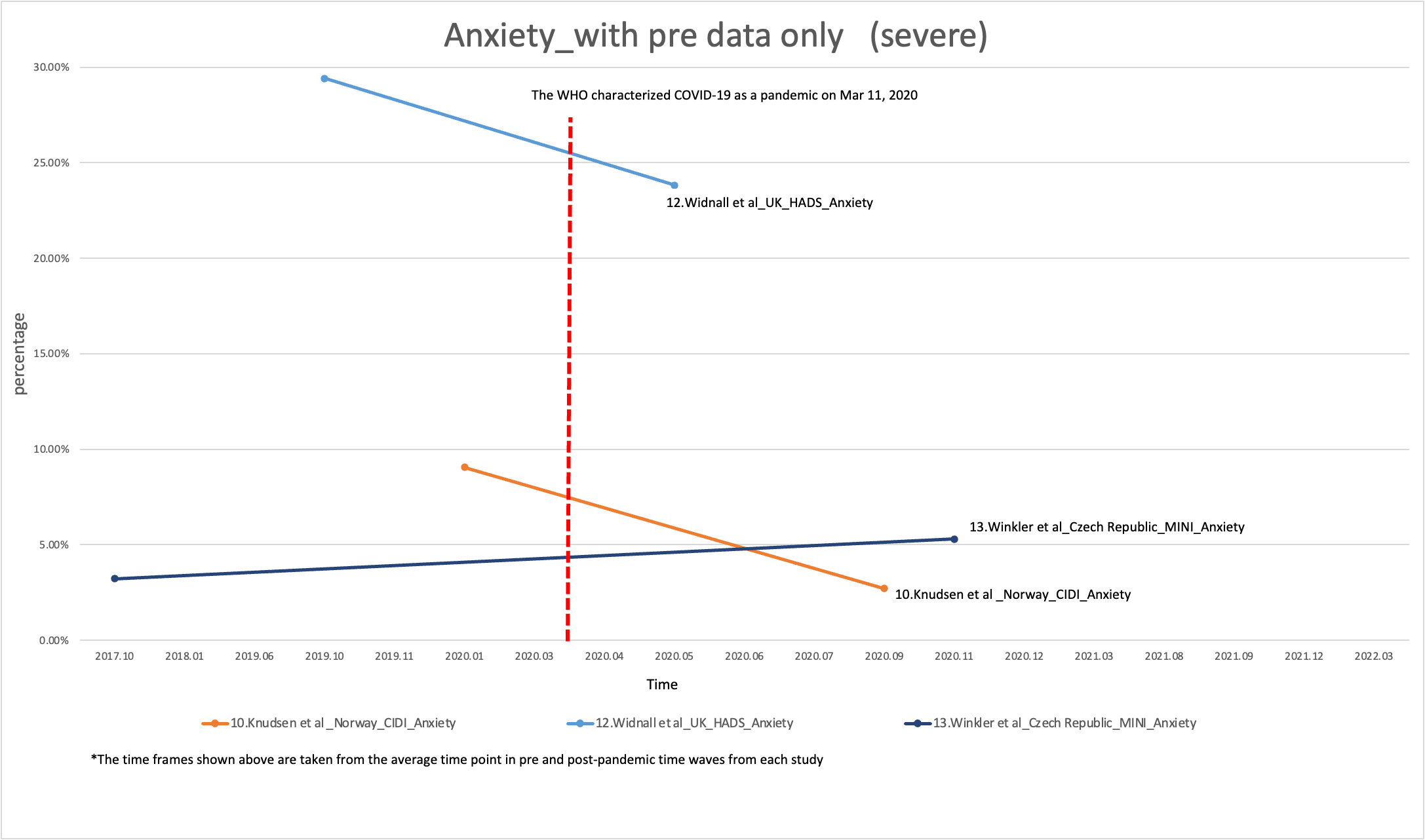


**Figure S1E:** Time plot graph of prevalence of mild depression from studies with pre-pandemic data


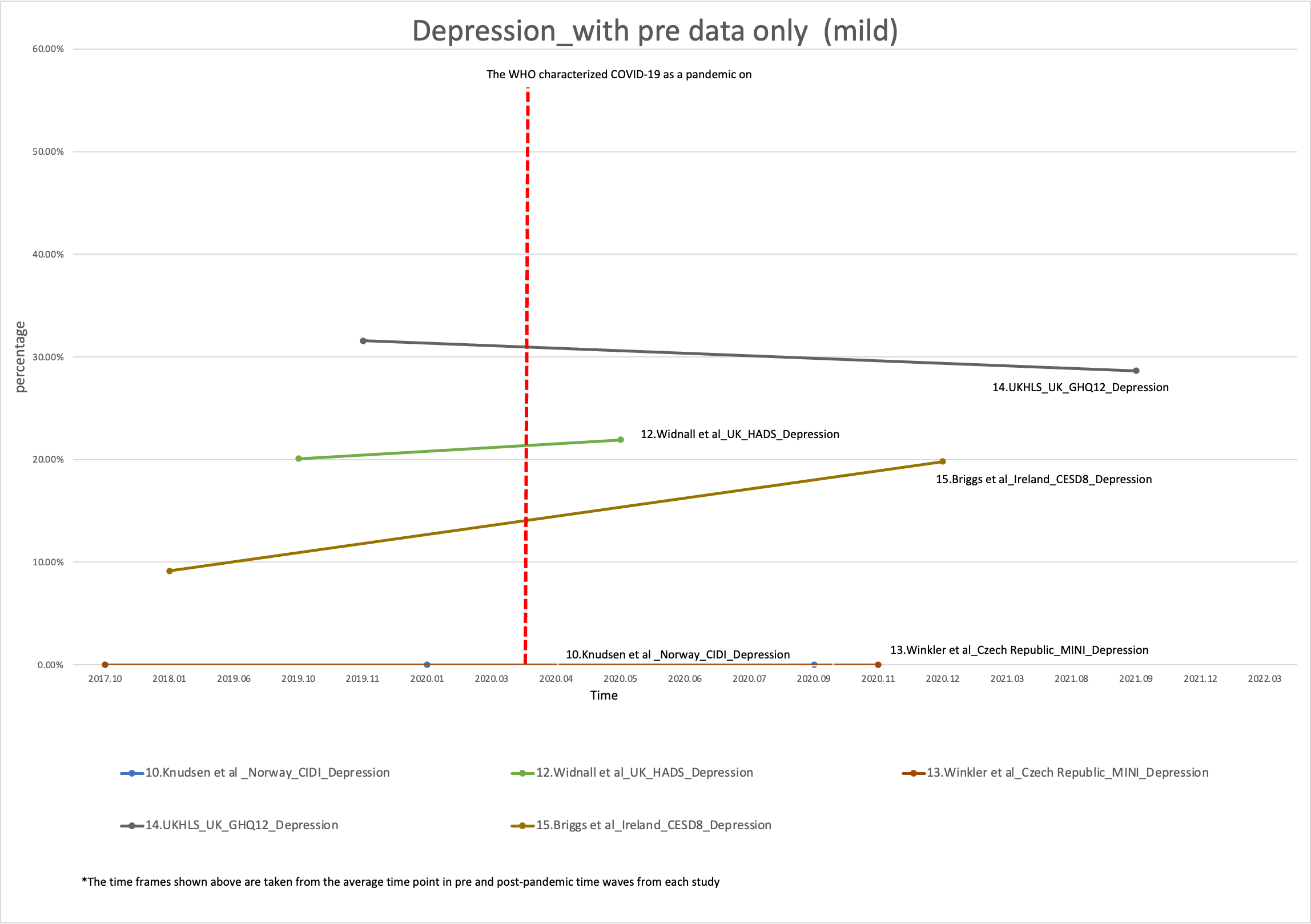


**Figure S1F:** Time plot graph of prevalence of severe depression from studies with pre-pandemic data


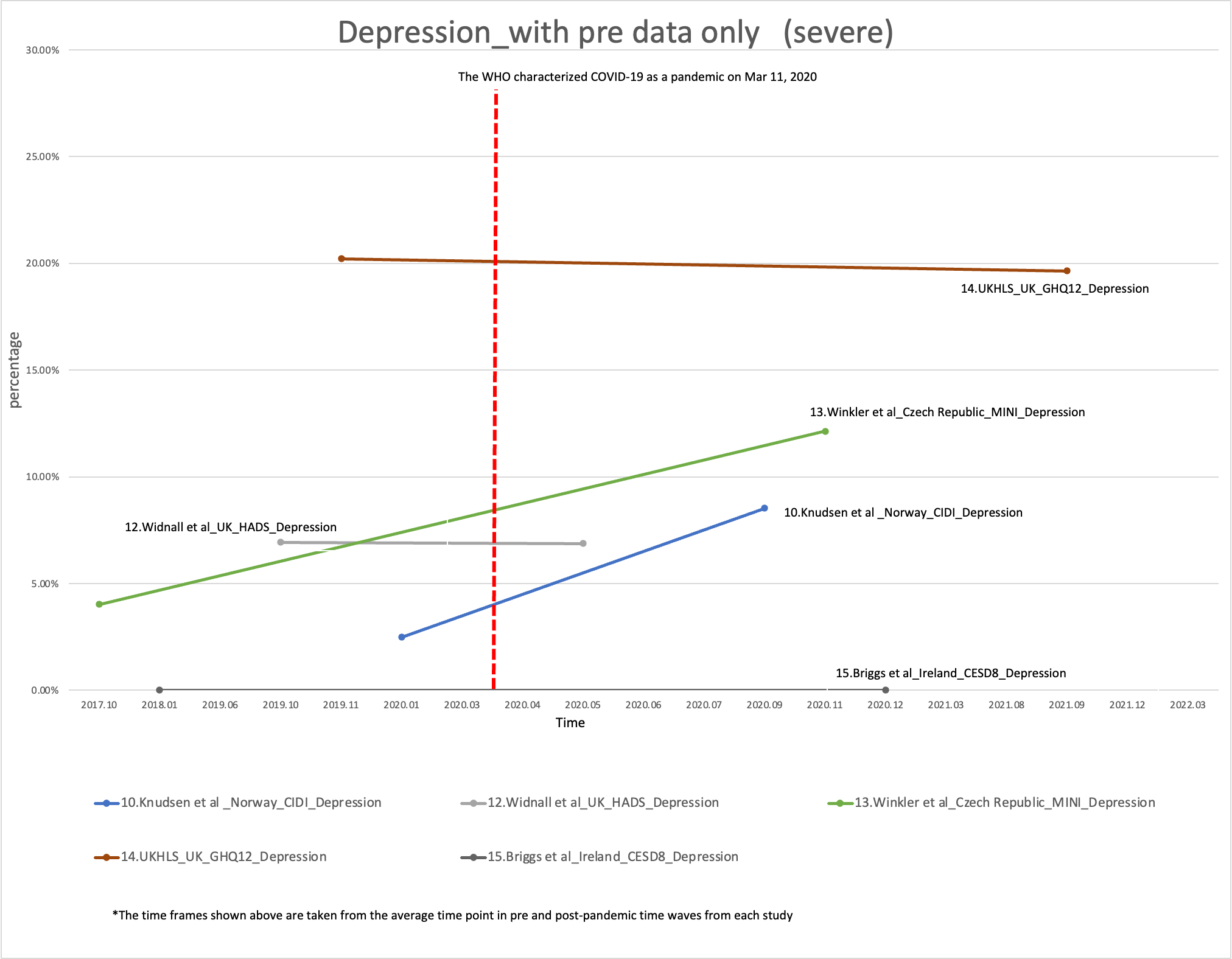


**Figure S2A:** Time plot graph of prevalence of mild depression and anxiety combined from studies with early pandemic data


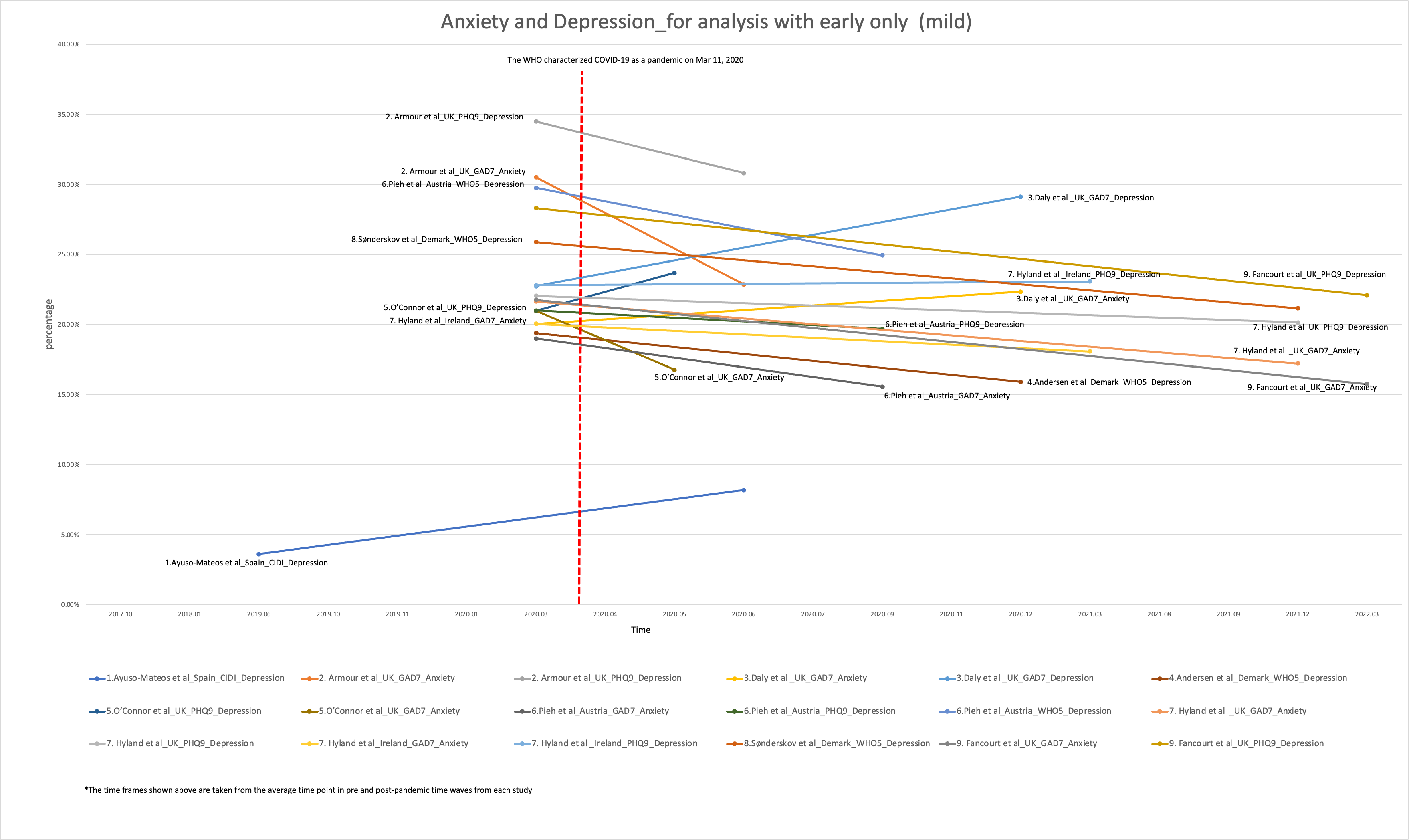


**Figure S2B:** Time plot graph of prevalence of severe depression and anxiety combined from studies with early pandemic data


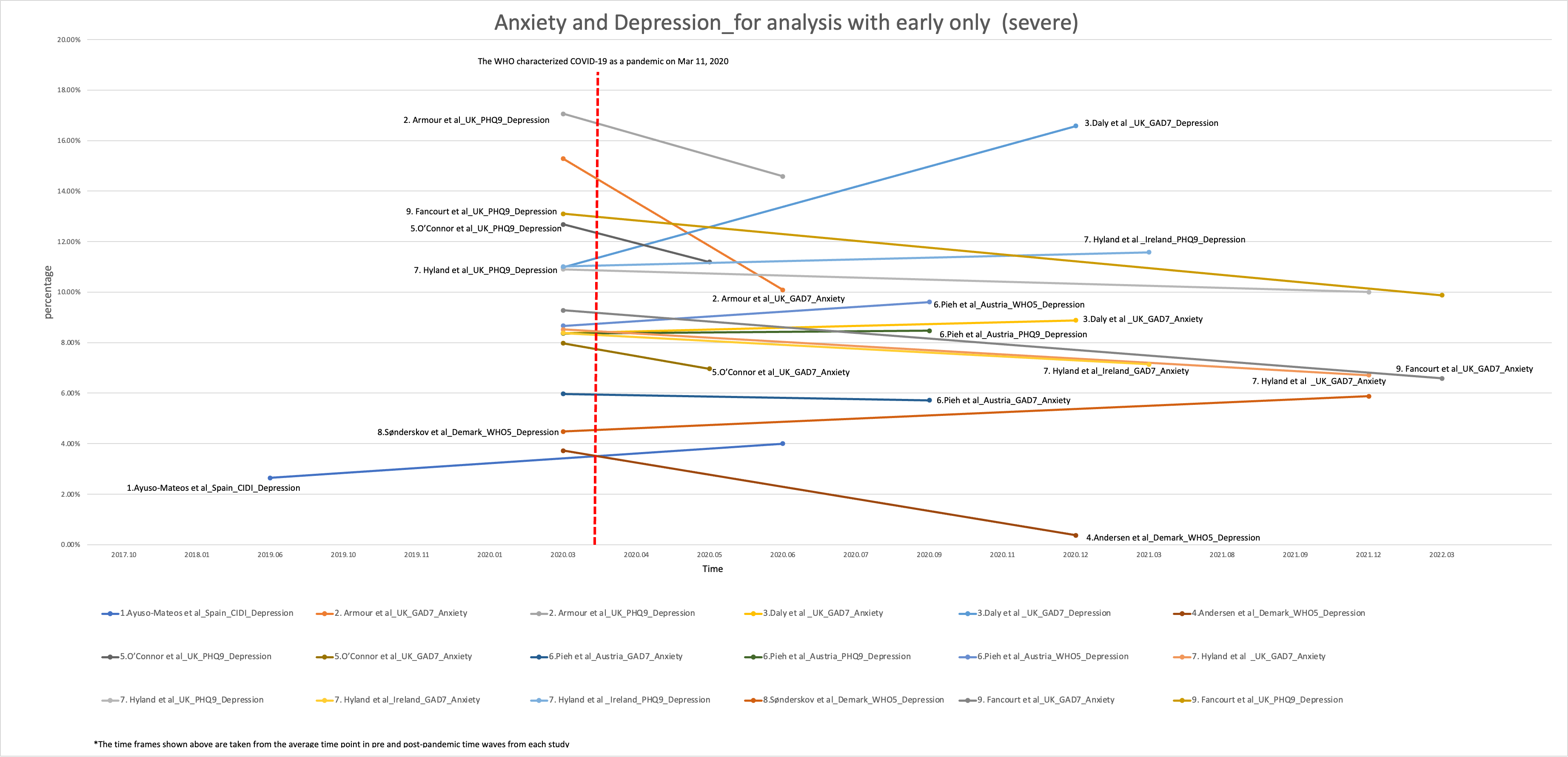


**Figure S2C:** Time plot graph of prevalence of mild anxiety from studies with early pandemic data


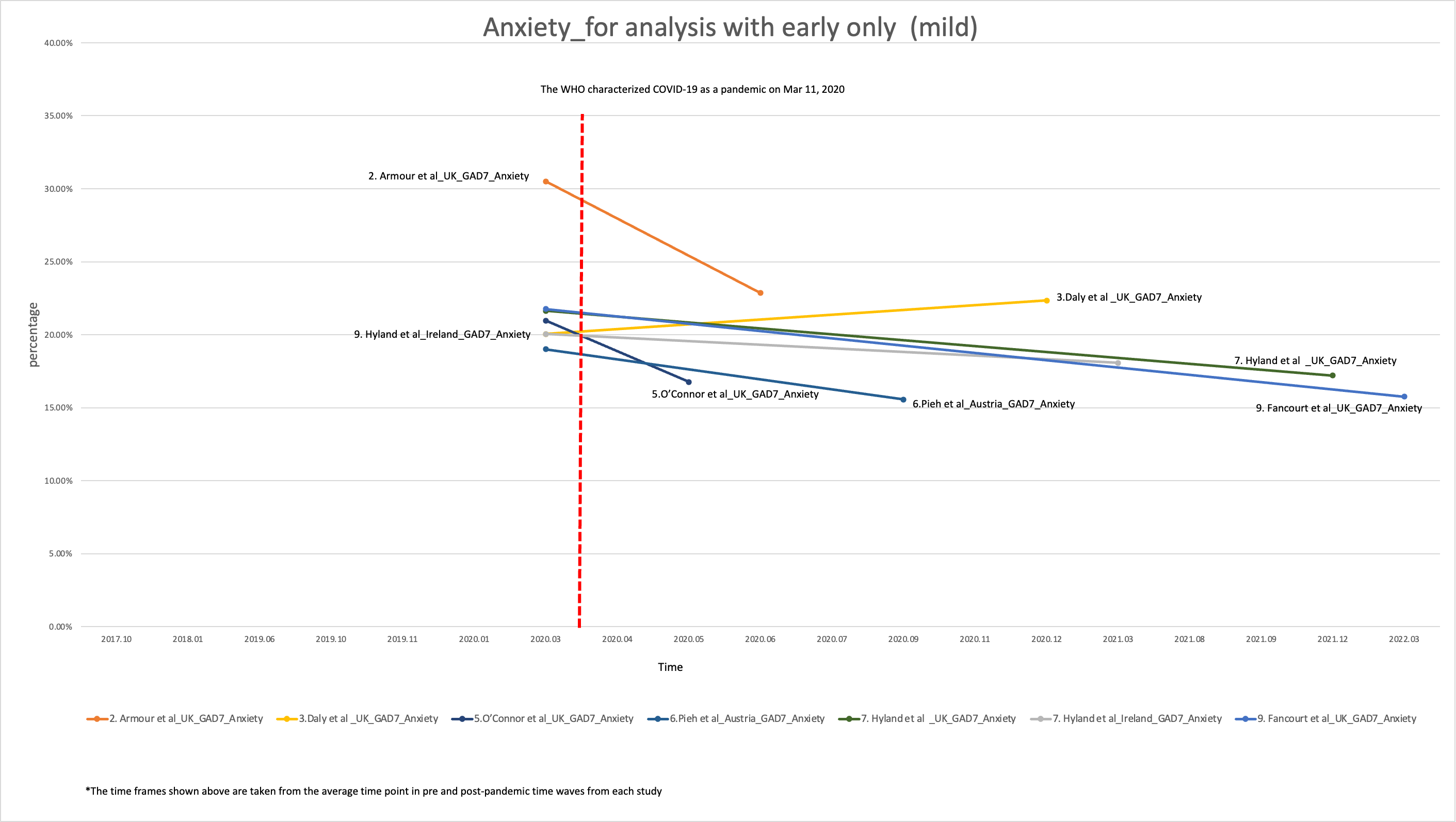


**Figure S2D:** Time plot graph of prevalence of severe anxiety from studies with early pandemic data


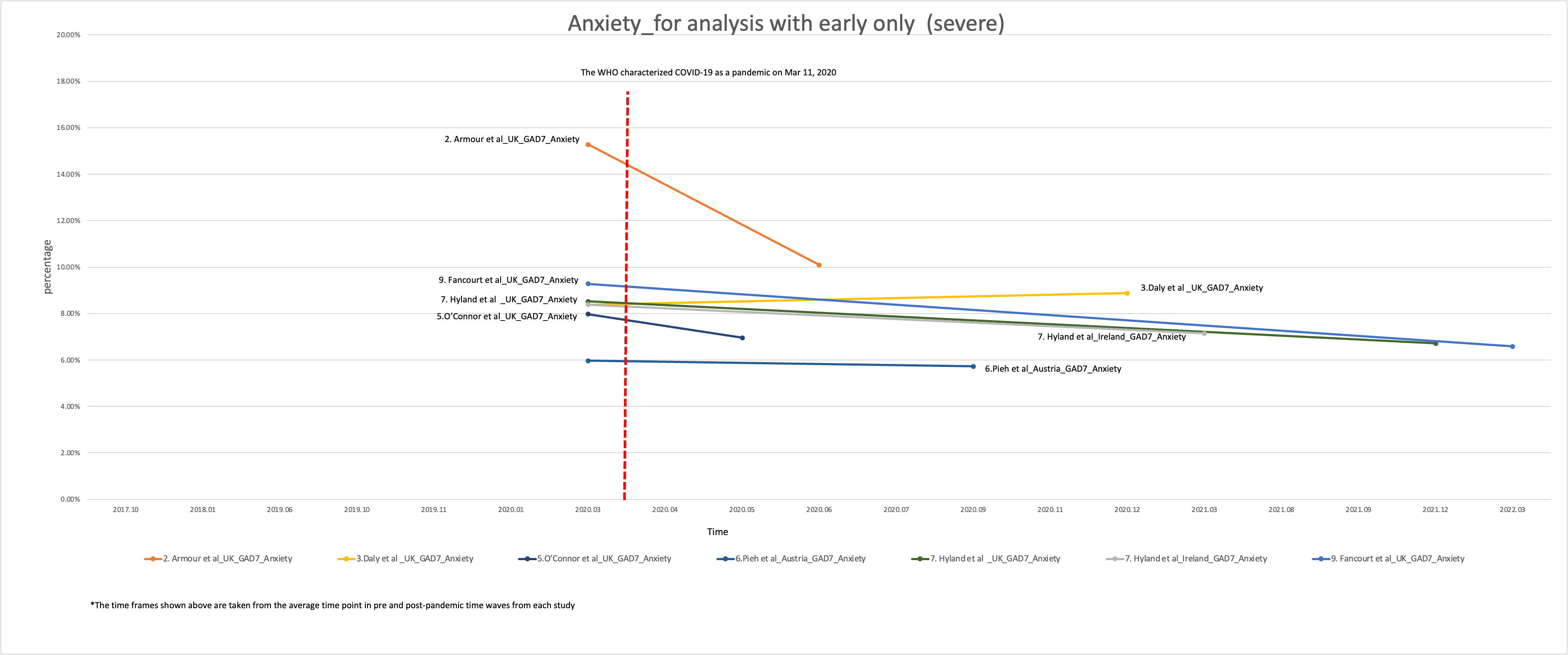


**Figure S2E:** Time plot graph of prevalence of mild depression from studies with early pandemic data


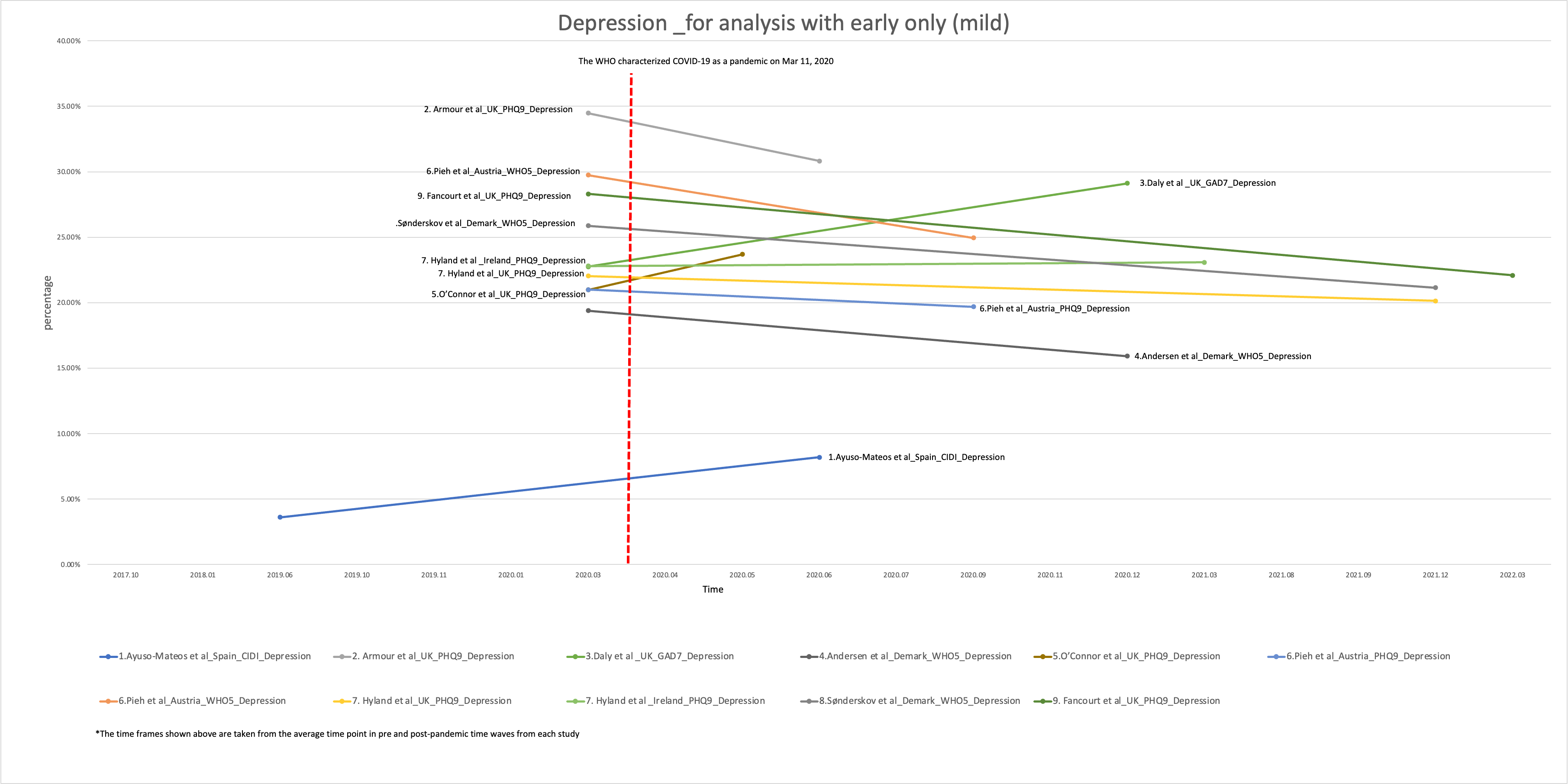


**Figure S2F:** Time plot graph of prevalence of severe depression from studies with early pandemic data


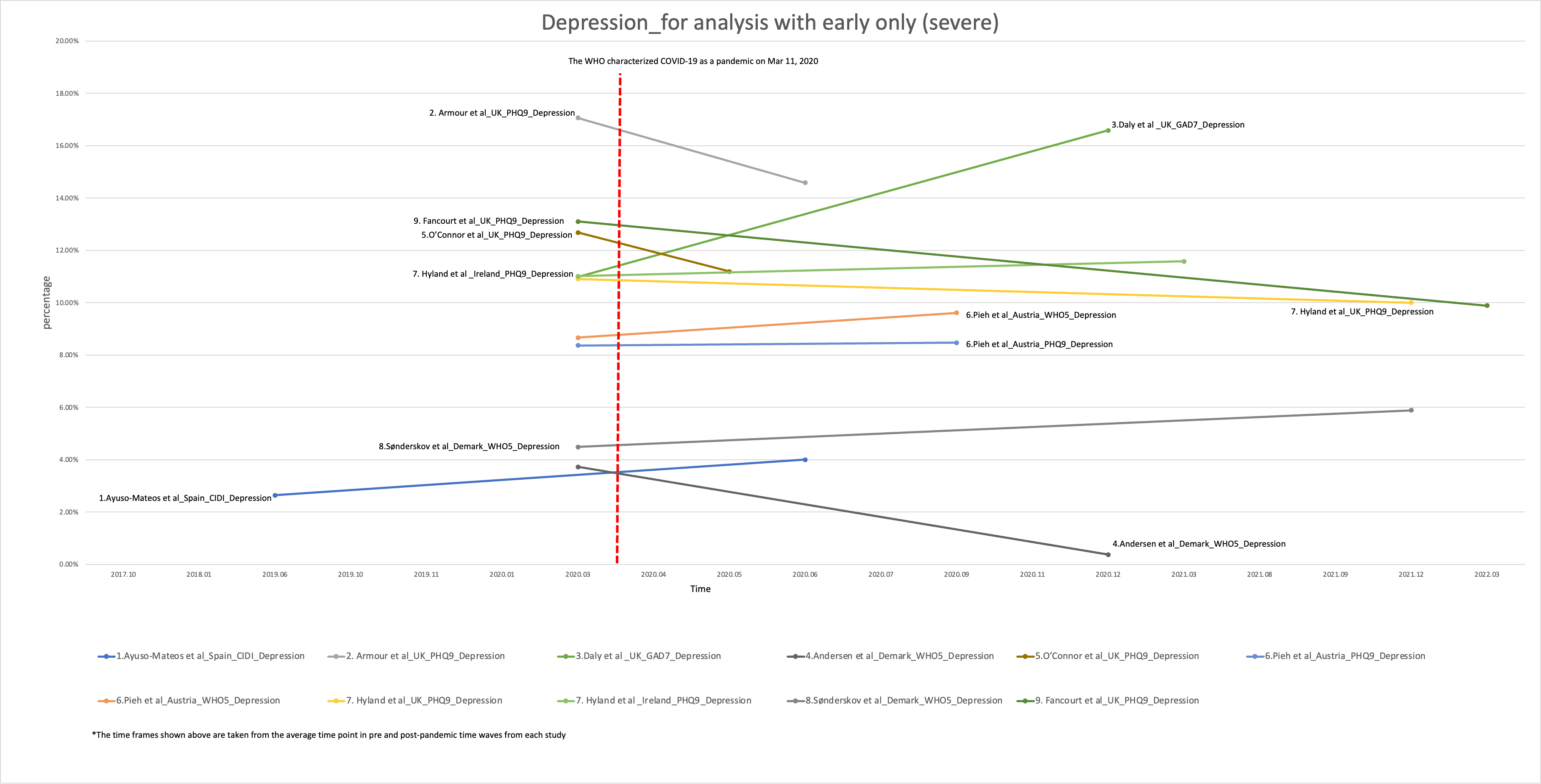


**Figure S3A.** Forest plot and Egger’s test for potential publication bias analyses on early to during-pandemic changes in prevalence of self-reported mild depression and anxiety combined

p-value = 0.17

**Figure S3B.** Forest plot and Egger’s test for potential publication bias analyses on early to during-pandemic changes in prevalence of self-reported mild anxiety alone

p-value = 0.14

**Figure S3C.** Forest plot and Egger’s test for potential publication bias analyses on early to during-pandemic changes in prevalence of self-reported mild depression alone

p-value = 0.74

**Figure S3D.** Forest plot and Egger’s test for potential publication bias analyses on early to during-pandemic changes in prevalence of self-reported severe depression and anxiety combined

p-value = 0.001

**Figure S3E.** Forest plot and Egger’s test for potential publication bias analyses on early to during-pandemic changes in prevalence of self-reported severe anxiety alone

p-value = 0.50

**Figure S3F.** Forest plot and Egger’s test for potential publication bias analyses on early to during-pandemic changes in prevalence of self-reported severe depression alone

p-value = 0.01

**Table S3G**. Forest plot and Egger’s test for potential publication bias analyses on pre- to during-pandemic changes in prevalence of self-reported mild depression and anxiety combined


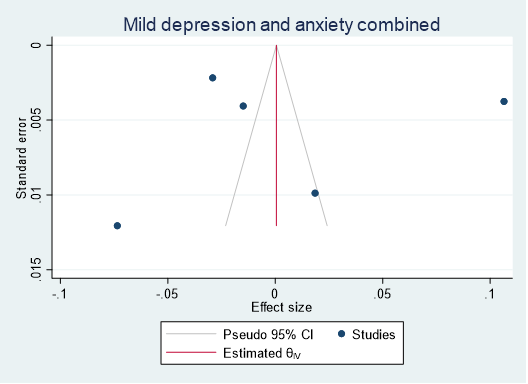


p-value = 0.49

**Table S3H**. Forest plot and Egger’s test for potential publication bias analyses on pre- to during-pandemic changes in prevalence of self-reported severe depression and anxiety combined


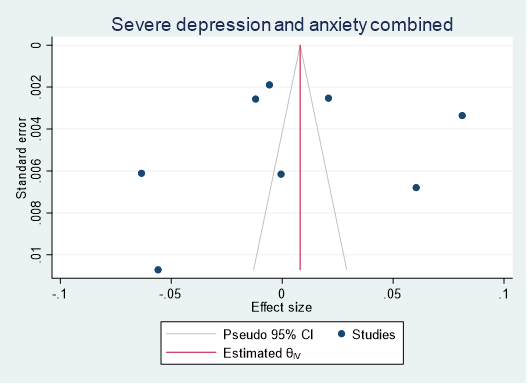


p-value = 0.20

**Figure S4A.** Trim-and-fill simulation technique result visualization for the outcomes with an indication of potential publication bias by the results of Egger’s test. Early to during-pandemic changes in prevalence of self-reported severe depression and anxiety combined.

**Note**: The use of non-parametric trim-and-fill method resulted in imputing 8 studies potential missing (denoted by squares) to the original collection of 18 studies (denoted by open circles). The overall pooled results suggested by trim-and-fill simulation technique (ΔPrev = -0.026 [95% CI: -0.035, -0.017]) were similar to the original results for this outcome. The indication of potentially missing studies could support the initial assumption that the asymmetry of the original funnel plot is due to publication bias.

**Figure S4B.** Trim-and-fill simulation technique result visualization for the outcomes with an indication of potential publication bias by the results of Egger’s test. Early to during-pandemic changes in prevalence of self-reported severe depression alone.

**Note**: The use of non-parametric trim-and-fill method resulted in imputing 6 studies potential missing (denoted by squares) to the original collection of 11 studies (denoted by open circles). The overall pooled results suggested by trim-and-fill simulation technique (ΔPrev = -0.028 [95%CI: -0.043, -0.013]) were slightly lower than the original results for this outcome. The indication of potentially missing studies could support the initial assumption that the asymmetry of the original funnel plot is due to publication bias.

**Note S5:**

Influence analyses (leave-one-out) for individual studies included into (A) pre-to-during pandemic and (B) early-to-during pandemic main analyses (using data for all sexes, all ages as 0-65+ years).

**A. For pre-to-during pandemic data**

**Due to a small number of studies, the influence analyses for pre-to-during pandemic data were conducted for studies on anxiety and depression combined (mild, and separately, severe).**

A1) Mild anxiety and depression combined. No individual study, if omitted, altered the observed non-significant pooled estimate.

--------------------------------------------------------------------------------------------------------------------

Study omitted | Estimate [95% Conf. Interval]

-----------------------------------------------------------------------+--------------------------------------------

11. van der Velden et al_Netherlands_MHI-5_Anxiety&depressive symptoms| -.00952258 -.14841528 .12937011

12. Widnall et al_UK_HADS_Anxiety | -.00849996 -.11010273 .09310281

12. Widnall et al_UK_HADS_Depression | -.01098368 -.11557413 .09360677

14. UKHLS_UK_GHQ-12_Depression | .03295222 -.06216435 .12806879

15. Briggs et al_Ireland_CESD-8_Depression | -.02699498 -.05683141 .00284145

-----------------------------------------------------------------------+---------------------------------------------

Combined | -.0102084 -.10872853 .08831172

---------------------------------------------------------------------------------------------------------------------

A2) Severe anxiety and depression combined. No individual study, if omitted, altered the observed non-significant pooled estimate.

--------------------------------------------------------------------------------------------------------------------

Study omitted | Estimate [95% Conf. Interval]

-----------------------------------------------------------------------+--------------------------------------------

10. Knudsen et al _Trondheim, Norway_CIDI_Anxiety | .00366186 -.04282906 .05015278

10. Knudsen et al _Trondheim, Norway_CIDI_Depression | .00151151 -.04715305 .05017608

11. van der Velden et al_Netherlands_MHI-5_Anxiety&depressive symptoms| .00426569 -.05613895 .06467032

12. Widnall et al_UK_HADS_Anxiety | .00382364 -.0453363 .05298357

12. Widnall et al_UK_HADS_Depression | .0024763 -.04867012 .05362273

13. Winkler et al_Czech Republic_MINI_Anxiety | .00061985 -.06040645 .06164614

13. Winkler et al_Czech Republic_MINI_Depression | -.00522 -.04029533 .02985533

14. UKHLS_UK_GHQ-12_Depression | .01616277 -.02595796 .05828351

-----------------------------------------------------------------------+--------------------------------------------

Combined | .00240492 -.04620546 .0510153

--------------------------------------------------------------------------------------------------------------------

**B. Early-to-during pandemic data**

B1) Mild anxiety and depression combined. The analysis showed that the largest studies in the collection (9. Fancourt at al., on depression measured with PHQ-9 and on anxiety measured with GAD-7) if admitted may marginally altered the observed pooled effect towards non-significant change in prevalence.

--------------------------------------------------------------------------------------------

Study omitted | Estimate [95% Conf. Interval]

---------------------------------------------+----------------------------------------------

1. Ayuso-Mateos et al_Spain_CIDI_Depression | -.05107424 -.08034594 -.02180254

2. Armour et al_UK_GAD-7_Anxiety | -.04936471 -.08585544 -.01287399

2. Armour et al_UK_PHQ-9_Depression | -.05015117 -.0868459 -.01345643

3. Daly et al _Ireland_GAD-7_Anxiety | -.05075898 -.08590386 -.01561411

3. Daly et al _Ireland_PHQ-9_Depression | -.05124496 -.0848461 -.01764382

4. Andersen et al_Denmark_WHO-5_Depression | -.05027004 -.08750676 -.01303331

5. O’Connor et al_UK_PHQ-9_Depression | -.0523864 -.08615621 -.01861658

5. O’Connor et al_UK_GAD-7_Anxiety | -.05014135 -.08789296 -.01238974

6. Pieh et al_Austria_GAD-7_Anxiety | -.050014 -.08613672 -.01389128

6. Pieh et al_Austria_PHQ-9_Depression | -.05018505 -.08611526 -.01425484

6. Pieh et al_Austria_WHO-5_Depression | -.04990439 -.08598059 -.0138282

7. Hyland et al _UK_GAD-7_Anxiety | -.04999755 -.08687316 -.01312193

7. Hyland et al _UK_PHQ-9_Depression | -.05048534 -.08701098 -.01395971

7. Hyland et al _Ireland_GAD-7_Anxiety | -.05025193 -.08645816 -.0140457

7. Hyland et al _Ireland_PHQ-9_Depression | -.05052002 -.08629213 -.01474791

8. Sønderskov et al_Denmark_WHO-5_Depression| -.04994719 -.08690646 -.01298793

9. Fancourt et al_UK_PHQ-9_Depression | -.04270942 -.09097364 .0055548

9. Fancourt et al_UK_GAD-7_Anxiety | -.04398623 -.09319682 .00522437

---------------------------------------------+----------------------------------------------

Combined | -.04988989 -.08546336 -.01431642

--------------------------------------------------------------------------------------------

B2) Mild anxiety alone. No influence from individual study appeared, since no omitted studies altered the observed significant reduction in pooled estimate.

---------------------------------------------------------------------------------------------

Study omitted | Estimate [95% Conf. Interval]

----------------------------------------------+----------------------------------------------

2. Armour et al_UK_GAD-7_Anxiety | -.05450566 -.09654262 -.0124687

3. Daly et al _Ireland_GAD-7_Anxiety | -.0574527 -.08206501 -.03284039

5. O’Connor et al_UK_GAD-7_Anxiety | -.05637795 -.10423505 -.00852084

6. Pieh et al_Austria_GAD-7_Anxiety | -.0557836 -.09599999 -.01556721

7. Hyland et al _UK_GAD-7_Anxiety | -.05588852 -.09966129 -.01211575

7. Hyland et al _Ireland_GAD-7_Anxiety | -.05635067 -.09518502 -.01751631

9. Fancourt et al._UK_GAD-7_Anxiety | -.03829702 -.06437677 -.01221726

----------------------------------------------+----------------------------------------------

Combined | -.05541859 -.09386504 -.01697214

---------------------------------------------------------------------------------------------

B3) Mild depression alone. No individual study, if omitted, altered the observed non-significant pooled estimate.

--------------------------------------------------------------------------------------------

Study omitted | Estimate [95% Conf. Interval]

----------------------------------------------+---------------------------------------------

1. Ayuso-Mateos et al_Spain_CIDI_Depression | -.04719467 -.10510012 .01071078

2. Armour et al_UK_PHQ-9_Depression | -.0453814 -.11759353 .02683073

3. Daly et al _Ireland_PHQ-9_Depression | -.04752061 -.11220144 .01716023

4. Andersen et al_Denmark_WHO-5_Depression | -.04556009 -.12093157 .02981138

5. O’Connor et al_UK_PHQ-9_Depression | -.04957515 -.11826204 .01911174

6. Pieh et al_Austria_PHQ-9_Depression | -.04554865 -.11485362 .02375632

6. Pieh et al_Austria_WHO-5_Depression | -.04501935 -.11430902 .02427031

7. Hyland et al _UK_PHQ-9_Depression | -.04602225 -.1185341 .02648959

7. Hyland et al _Ireland_PHQ-9_Depression | -.04614829 -.1157452 .02344862

8. Sønderskov et al_Denmark_WHO-5_Depression | -.04497252 -.11831767 .02837263

9. Fancourt et al._UK_PHQ-9_Depression | -.00732671 -.03471543 .02006202

----------------------------------------------+---------------------------------------------

Combined | -.04506511 -.11222808 .02209787

--------------------------------------------------------------------------------------------

B4) Severe anxiety and depression combined. The analysis showed that the largest studies in the collection (9. Fancourt at al., on depression measured with PHQ-9 and on anxiety measured with GAD-7) if admitted may marginally altered the observed pooled effect towards non-significant change in prevalence.

-------------------------------------------------------------------------------------------

Study omitted | Estimate [95% Conf. Interval]

---------------------------------------------+----------------------------------------------

1. Ayuso-Mateos et al_Spain_CIDI_Depression | -.02455842 -.04130279 -.00781406

2. Armour et al_UK_PHQ-9_Depression | -.0240797 -.04241112 -.00574827

2. Armour et al_UK_GAD-7_Anxiety | -.02354456 -.04143841 -.00565072

3. Daly et al _Ireland_PHQ-9_Depression | -.02504773 -.04113192 -.00896354

3. Daly et al _Ireland_GAD-7_Anxiety | -.02444118 -.04227513 -.00660722

4. Andersen et al_Denmark_WHO-5_Depression | -.02385637 -.0429296 -.00478314

5. O’Connor et al_UK_PHQ-9_Depression | -.02439399 -.04305312 -.00573485

5. O’Connor et al_UK_GAD-7_Anxiety | -.02454578 -.04320853 -.00588303

6. Pieh et al_Austria_PHQ-9_Depression | -.02429671 -.04219477 -.00639865

6. Pieh et al_Austria_GAD-7_Anxiety | -.02426795 -.0422112 -.0063247

6. Pieh et al_Austria_WHO-5_Depression | -.02436466 -.04211786 -.00661146

7. Hyland et al _UK_PHQ-9_Depression | -.02438583 -.04265213 -.00611953

7. Hyland et al _UK_GAD-7_Anxiety | -.02421087 -.04265805 -.00576368

7. Hyland et al _Ireland_PHQ-9_Depression | -.02445095 -.04231455 -.00658736

7. Hyland et al _Ireland_GAD-7_Anxiety | -.0242341 -.04240451 -.00606369

8. Sønderskov et al_Denmark_WHO-5_Depression| -.02492972 -.04147862 -.00838081

9. Fancourt et al._UK_GAD-7_Anxiety | -.0223811 -.04728309 .0025209

9. Fancourt et al._UK_PHQ-9_Depression | -.01944405 -.04230733 .00341924

---------------------------------------------+----------------------------------------------

Combined | -.02409439 -.04190279 -.006286

--------------------------------------------------------------------------------------------

B5) Severe anxiety alone. No influence from individual study appeared, since no omitted studies altered the observed significant reduction in pooled estimate.

--------------------------------------------------------------------------------------------

Study omitted | Estimate [95% Conf. Interval]

---------------------------------------------+----------------------------------------------

2. Armour et al_UK_GAD-7_Anxiety | -.02383457 -.04421364 -.00345549

3. Daly et al _Ireland_GAD-7_Anxiety | -.02578036 -.0457327 -.00582802

5. O’Connor et al_UK_GAD-7_Anxiety | -.02607384 -.05085906 -.00128862

6. Pieh et al_Austria_GAD-7_Anxiety | -.02539407 -.04690064 -.0038875

7. Hyland et al _UK_GAD-7_Anxiety | -.02529633 -.05004698 -.00054567

7. Hyland et al _Ireland_GAD-7_Anxiety | -.02533005 -.04844693 -.00221317

9. Fancourt et al._UK_GAD-7_Anxiety | -.0175248 -.03287704 -.00217257

---------------------------------------------+-----------------------------------------------

Combined | -.02500237 -.04667462 -.00333011

---------------------------------------------------------------------------------------------

B6) Severe depression alone. No studies, if omitted, altered the observed non-significant pooled estimate.

---------------------------------------------------------------------------------------------

Study omitted | Estimate [95% Conf. Interval]

---------------------------------------------+-----------------------------------------------

1. Ayuso-Mateos et al_Spain_CIDI_Depression | -.02416197 -.05561812 .00729417

2. Armour et al_UK_PHQ-9_Depression | -.0232442 -.05763837 .01114998

3. Daly et al _Ireland_PHQ-9_Depression | -.02508821 -.05426212 .0040857

4. Andersen et al_Denmark_WHO-5_Depression | -.022808 -.06156631 .01595032

5. O’Connor et al_UK_PHQ-9_Depression | -.02382994 -.05975523 .01209535

6. Pieh et al_Austria_PHQ-9_Depression | -.02367157 -.05678092 .00943777

6. Pieh et al_Austria_WHO-5_Depression | -.02379971 -.05658308 .00898366

7. Hyland et al _UK_PHQ-9_Depression | -.02382805 -.05828475 .01062865

7. Hyland et al _Ireland_PHQ-9_Depression | -.02395885 -.05713003 .00921233

8. Sønderskov et al_Denmark_WHO-5_Depression| -.02486366 -.05624817 .00652084

9. Fancourt et al._UK_PHQ-9_Depression | -.00396951 -.02145899 .01351996

---------------------------------------------+------------------------------------------------

Combined | -.02330202 -.05572054 .00911649

----------------------------------------------------------------------------------------------

**References:**

1. Robins LN, Wing J, Wittchen HU, Helzer JE, Babor TF, Burke J, et al. The Composite International Diagnostic Interview. An epidemiologic Instrument suitable for use in conjunction with different diagnostic systems and in different cultures. Arch Gen Psychiatry. 1988;45(12):1069-77.

2. Spitzer RL, Kroenke K, Williams JB, Lowe B. A brief measure for assessing generalized anxiety disorder: the GAD-7. Arch Intern Med. 2006;166(10):1092-7.

3. Zigmond AS, Snaith RP. The hospital anxiety and depression scale. Acta Psychiatr Scand. 1983;67(6):361-70.

4. Rumpf HJ, Meyer C, Hapke U, John U. Screening for mental health: validity of the MHI-5 using DSM-IV Axis I psychiatric disorders as gold standard. Psychiatry Res. 2001;105(3):243-53.

5. Kroenke K, Spitzer RL, Williams JB. The PHQ-9: validity of a brief depression severity measure. J Gen Intern Med. 2001;16(9):606-13.

6. WHO. Wellbeing Measures in Primary Health Care/The Depcare Project. Copenhagen: WHO Regional Office for Europe; 1998.

7. Kubinec R, Barcelo J, Goldszmidt R, Grujic V, Model T, Schenk C, et al. Cross-National Measures of the Intensity of COVID-19 Public Health Policies. May 1 ed. SocArXiv Papers2021.
