# Supplementary material for: Changes in anxiety and depression during the COVID-19 pandemic in the European population: A meta-analysis of changes and associations with restriction policies
Source: Eur Psychiatry. 2023 Oct 26;66(1):e87. doi: 10.1192/j.eurpsy.2023.2467 (PMC10755582; doi:10.1192/j.eurpsy.2023.2467)
Supplement: Lok et al. supplementary material 2 — Lok et al. supplementary material [file S0924933823024677sup002.docx]

Appendix 2 Stata do-files for analysis:

**mild*

*baseline

gen baseline_var =(baseline*(1-baseline))

gen baseline_lowerCI= baseline-(1.96*sqrt(baseline_var/n_pre))

gen baseline_upperCI= baseline+(1.96*sqrt(baseline_var/n_pre))

*final

gen final_var = (final*(1-final))

gen final_lowerCI= final-(1.96*sqrt(final_var/n_post))

gen final_upperCI= final+(1.96*sqrt(final_var/n_post))

*pool

gen pool_var = ((baseline_var*(n_pre-1))/(n_pre+n_post))+((final_var *(n_post-1))/(n_pre+n_post))

*additional ci

gen additional = final-baseline

gen additional_lowerCI = (final-baseline)-(1.96*sqrt((pool_var)/(n_post+n_pre)))

gen additional_upperCI = (final-baseline)+(1.96*sqrt((pool_var)/(n_post+n_pre)))

metan additional additional_lowerCI additional_upperCI, wgt(sum_n) label(namevar==Study) random title(Anxiety and Depression (mild))

metan additional additional_lowerCI additional_upperCI, wgt(sum_n) by(Country) label(namevar==Study) random title(Anxiety and Depression (mild) by country)

*severe

*baseline

gen severe_baseline_var =(severe_baseline*(1-severe_baseline))

gen severe_baseline_lowerCI= severe_baseline-(1.96*sqrt(severe_baseline_var)/severe_n_pre)

gen severe_baseline_upperCI= severe_baseline+(1.96*sqrt(severe_baseline_var)/severe_n_pre)

*final

gen severe_final_var = (severe_final*(1-severe_final))

gen severe_final_lowerCI= severe_final-(1.96*sqrt(severe_final_var)/severe_n_post)

gen severe_final_upperCI= severe_final+(1.96*sqrt(severe_final_var)/severe_n_post)

*pool

gen severe_pool_var = ((severe_baseline_var*(severe_n_pre-1))/(severe_n_pre+severe_n_post))+((severe_final_var *(severe_n_post-1))/(severe_n_pre+severe_n_post))

*additional ci

gen severe_additional = severe_final-severe_baseline

gen severe_additional_lowerCI = (severe_final-severe_baseline)-(1.96*sqrt((severe_pool_var)/(severe_n_post+severe_n_pre)))

gen severe_additional_upperCI = (severe_final-severe_baseline)+(1.96*sqrt((severe_pool_var)/(severe_n_post+severe_n_pre)))

*change in %

gen severe_additionalinper = severe_additional*0.1

metan severe_additional severe_additional_lowerCI severe_additional_upperCI, wgt(sum_n) label(namevar==Study) random title(Anxiety and Depression (severe))

metan severe_additional severe_additional_lowerCI severe_additional_upperCI, wgt(sum_n) by(Country) label(namevar==Study) random title(Anxiety and Depression (severe) by country)

clear

*rq1 depression

list Study n_post n_pre

**mild*

*baseline

gen baseline_var =(baseline*(1-baseline))

gen baseline_lowerCI= baseline-(1.96*sqrt(baseline_var/n_pre))

gen baseline_upperCI= baseline+(1.96*sqrt(baseline_var/n_pre))

*final

gen final_var = (final*(1-final))

gen final_lowerCI= final-(1.96*sqrt(final_var/n_post))

gen final_upperCI= final+(1.96*sqrt(final_var/n_post))

*pool

gen pool_var = ((baseline_var*(n_pre-1))/(n_pre+n_post))+((final_var *(n_post-1))/(n_pre+n_post))

*additional ci

gen additional = final-baseline

gen additional_lowerCI = (final-baseline)-(1.96*sqrt((pool_var)/(n_post+n_pre)))

gen additional_upperCI = (final-baseline)+(1.96*sqrt((pool_var)/(n_post+n_pre)))

*change in %

gen additionalinper = additional*0.1

metan additional additional_lowerCI additional_upperCI, wgt(sum_n) label(namevar==Study) random title(Depression (mild))

metan additional additional_lowerCI additional_upperCI, wgt(sum_n) by(Country) label(namevar==Study) random title(Depression (mild) by country)

*severe

*baseline

gen severe_baseline_var =(severe_baseline*(1-severe_baseline))

gen severe_baseline_lowerCI= severe_baseline-(1.96*sqrt(severe_baseline_var)/severe_n_pre)

gen severe_baseline_upperCI= severe_baseline+(1.96*sqrt(severe_baseline_var)/severe_n_pre)

*final

gen severe_final_var = (severe_final*(1-severe_final))

gen severe_final_lowerCI= severe_final-(1.96*sqrt(severe_final_var)/severe_n_post)

gen severe_final_upperCI= severe_final+(1.96*sqrt(severe_final_var)/severe_n_post)

*pool

gen severe_pool_var = ((severe_baseline_var*(severe_n_pre-1))/(severe_n_pre+severe_n_post))+((severe_final_var *(severe_n_post-1))/(severe_n_pre+severe_n_post))

*additional ci

gen severe_additional = severe_final-severe_baseline

gen severe_additional_lowerCI = (severe_final-severe_baseline)-(1.96*sqrt((severe_pool_var)/(severe_n_post+severe_n_pre)))

gen severe_additional_upperCI = (severe_final-severe_baseline)+(1.96*sqrt((severe_pool_var)/(severe_n_post+severe_n_pre)))

*change in %

gen severe_additionalinper = severe_additional*0.1

metan severe_additional severe_additional_lowerCI severe_additional_upperCI, wgt(sum_n) label(namevar==Study) random title(Depression (severe))

metan severe_additional severe_additional_lowerCI severe_additional_upperCI, wgt(sum_n) by(Country) label(namevar==Study) random title(Depression (severe) by country)

**mild*

*baseline

gen baseline_var =(baseline*(1-baseline))

gen baseline_lowerCI= baseline-(1.96*sqrt(baseline_var)/n_pre)

gen baseline_upperCI= baseline+(1.96*sqrt(baseline_var)/n_pre)

*final

gen final_var = (final*(1-final))

gen final_lowerCI= final-(1.96*sqrt(final_var)/n_post)

gen final_upperCI= final+(1.96*sqrt(final_var)/n_post)

*pool

gen pool_var = ((baseline_var*(n_pre-1))/(n_pre+n_post))+((final_var *(n_post-1))/(n_pre+n_post))

*additional ci

gen additional = final-baseline

gen additional_lowerCI = (final-baseline)-(1.96*sqrt((pool_var)/(n_post+n_pre)))

gen additional_upperCI = (final-baseline)+(1.96*sqrt((pool_var)/(n_post+n_pre)))

*change in %

gen additionalinper = additional*0.1

metan additional additional_lowerCI additional_upperCI, wgt(sum_n) label(namevar==Study) random title(Anxiety (mild))

metan additional additional_lowerCI additional_upperCI, wgt(sum_n) by(Country) label(namevar==Study) random title(Anxiety (mild) by country)

*severe

*baseline

gen severe_baseline_var =(severe_baseline*(1-severe_baseline))

gen severe_baseline_lowerCI= severe_baseline-(1.96*sqrt(severe_baseline_var)/severe_n_pre)

gen severe_baseline_upperCI= severe_baseline+(1.96*sqrt(severe_baseline_var)/severe_n_pre)

*final

gen severe_final_var = (severe_final*(1-severe_final))

gen severe_final_lowerCI= severe_final-(1.96*sqrt(severe_final_var)/severe_n_post)

gen severe_final_upperCI= severe_final+(1.96*sqrt(severe_final_var)/severe_n_post)

*pool

gen severe_pool_var = ((severe_baseline_var*(severe_n_pre-1))/(severe_n_pre+severe_n_post))+((severe_final_var *(severe_n_post-1))/(severe_n_pre+severe_n_post))

*additional ci

gen severe_additional = severe_final-severe_baseline

gen severe_additional_lowerCI = (severe_final-severe_baseline)-(1.96*sqrt((severe_pool_var)/(severe_n_post+severe_n_pre)))

gen severe_additional_upperCI = (severe_final-severe_baseline)+(1.96*sqrt((severe_pool_var)/(severe_n_post+severe_n_pre)))

*change in %

gen severe_additionalinper = severe_additional*0.1

metan severe_additional severe_additional_lowerCI severe_additional_upperCI, wgt(sum_n) label(namevar==Study) random title(Anxiety (severe))

metan severe_additional severe_additional_lowerCI severe_additional_upperCI, wgt(sum_n) by(Country) label(namevar==Study) random title(Anxiety (severe) by country)

clear

*rq2

**mild*

*baseline

gen baseline_var =(baseline*(1-baseline))

gen baseline_lowerCI= baseline-(1.96*sqrt(baseline_var)/n_pre)

gen baseline_upperCI= baseline+(1.96*sqrt(baseline_var)/n_pre)

*final

gen final_var = (final*(1-final))

gen final_lowerCI= final-(1.96*sqrt(final_var)/n_post)

gen final_upperCI= final+(1.96*sqrt(final_var)/n_post)

*pool

gen pool_var = ((baseline_var*(n_pre-1))/(n_pre+n_post))+((final_var *(n_post-1))/(n_pre+n_post))

*additional ci

gen additional = final-baseline

gen additional_lowerCI = (final-baseline)-(1.96*sqrt((pool_var)/(n_post+n_pre)))

gen additional_upperCI = (final-baseline)+(1.96*sqrt((pool_var)/(n_post+n_pre)))

*change in %

gen additionalinper = additional*0.1

*severe

*baseline

gen severe_baseline_var =(severe_baseline*(1-severe_baseline))

gen severe_baseline_lowerCI= severe_baseline-(1.96*sqrt(severe_baseline_var)/severe_n_pre)

gen severe_baseline_upperCI= severe_baseline+(1.96*sqrt(severe_baseline_var)/severe_n_pre)

*final

gen severe_final_var = (severe_final*(1-severe_final))

gen severe_final_lowerCI= severe_final-(1.96*sqrt(severe_final_var)/severe_n_post)

gen severe_final_upperCI= severe_final+(1.96*sqrt(severe_final_var)/severe_n_post)

*pool

gen severe_pool_var = ((severe_baseline_var*(severe_n_pre-1))/(severe_n_pre+severe_n_post))+((severe_final_var *(severe_n_post-1))/(severe_n_pre+severe_n_post))

*additional ci

gen severe_additional = severe_final-severe_baseline

gen severe_additional_lowerCI = (severe_final-severe_baseline)-(1.96*sqrt((severe_pool_var)/(severe_n_post+severe_n_pre)))

gen severe_additional_upperCI = (severe_final-severe_baseline)+(1.96*sqrt((severe_pool_var)/(severe_n_post+severe_n_pre)))

*change in %

gen severe_additionalinper = severe_additional*0.1

*sex

* mild

metan additional additional_lowerCI additional_upperCI if Sex=="1" & age=="0-65+" , label(namevar==Study) graphregion(color(white)) name(g1,replace) random title("Anxiety and Depression in male(mild)") wgt(sum_n)

metan additional additional_lowerCI additional_upperCI if Sex=="2" & age=="0-65+", label(namevar==Study) graphregion(color(white)) name(g2,replace) random title(" Anxiety and Depression in female(mild)" ) wgt(sum_n)

*sex severe

metan severe_additional severe_additional_lowerCI severe_additional_upperCI, by(sex) random wgt(sum_n)

metan severe_additional severe_additional_lowerCI severe_additional_upperCI if Sex=="1" & age=="0-65+" , label(namevar==Study) graphregion(color(white)) name(g1,replace) random title( Anxiety and Depression among male (severe)) wgt(sum_n)

metan severe_additional severe_additional_lowerCI severe_additional_upperCI if Sex=="2" & age=="0-65+" , label(namevar==Study) graphregion(color(white)) name(g2,replace) random title( Anxiety and Depression among female (severe)) wgt(sum_n)

metan severe_additional severe_additional_lowerCI severe_additional_upperCI, by(sex) random wgt(sum_n)

*age

*mild

metan additional additional_lowerCI additional_upperCI if Sex=="1_2" & age=="0-18" , label(namevar==Study) graphregion(color(white)) name(g1,replace) random title( Anxiety and Depression by aged 0-18(mild) ) wgt(sum_n)

metan additional additional_lowerCI additional_upperCI if Sex=="1_2" & age=="19-64", label(namevar==Study) graphregion(color(white)) name(g2,replace) random title( Anxiety and Depression by aged 19-64(mild) ) wgt(sum_n)

metan additional additional_lowerCI additional_upperCI if Sex=="1_2" & age=="65+", label(namevar==Study) graphregion(color(white)) name(g3,replace) random title( Anxiety and Depression by aged 65+(mild) ) wgt(sum_n)

metan additional additional_lowerCI additional_upperCI if Sex=="1_2" & age=="0-65+", label(namevar==Study) graphregion(color(white)) name(g4,replace) random title( Anxiety and Depression by aged 0-65+(mild) ) wgt(sum_n)

*severe

metan severe_additional severe_additional_lowerCI severe_additional_upperCI if Sex=="1_2" & age=="0-18" , label(namevar==Study) graphregion(color(white)) name(g1,replace) random title( Anxiety and Depression by aged 0-18(severe) ) wgt(sum_n)

metan severe_additional severe_additional_lowerCI severe_additional_upperCI if Sex=="1_2" & age=="19-64", label(namevar==Study) graphregion(color(white)) name(g2,replace) random title( Anxiety and Depression by aged 19-64(severe) ) wgt(sum_n)

metan severe_additional severe_additional_lowerCI severe_additional_upperCI if Sex=="1_2" & age=="65+", label(namevar==Study) graphregion(color(white)) name(g3,replace) random title( Anxiety and Depression by aged 65+(severe) ) wgt(sum_n)

metan severe_additional severe_additional_lowerCI severe_additional_upperCI if Sex=="1_2" & age=="0-65+", label(namevar==Study) graphregion(color(white)) name(g4,replace) random title(Anxiety and Depression by aged 0-65+(severe) ) wgt(sum_n)

*agexsex

*mild

metan additional additional_lowerCI additional_upperCI if Sex=="1" & age=="0-18" , label(namevar==Study) graphregion(color(white)) name(g1,replace) random title( Anxiety and Depression by male aged 0-18(mild) ) wgt(sum_n)

metan additional additional_lowerCI additional_upperCI if Sex=="1" & age=="19-64", label(namevar==Study) graphregion(color(white)) name(g2,replace) random title( Anxiety and Depression by male aged 19-64(mild) ) wgt(sum_n)

metan additional additional_lowerCI additional_upperCI if Sex=="1" & age=="65+", label(namevar==Study) graphregion(color(white)) name(g3,replace) random title(Anxiety and Depression by male aged 65+(mild) ) wgt(sum_n)

metan additional additional_lowerCI additional_upperCI if Sex=="1" & age=="0-65+", label(namevar==Study) graphregion(color(white)) name(g4,replace) random title( Anxiety and Depression by male aged 0-65+(mild) ) wgt(sum_n)

metan additional additional_lowerCI additional_upperCI if Sex=="2" & age=="0-18" , label(namevar==Study) graphregion(color(white)) name(g5,replace) random title( Anxiety and Depression by female aged 0-18(mild) ) wgt(sum_n)

metan additional additional_lowerCI additional_upperCI if Sex=="2" & age=="19-64", label(namevar==Study) graphregion(color(white)) name(g6,replace) random title( Anxiety and Depression by female aged 19-64(mild) ) wgt(sum_n)

metan additional additional_lowerCI additional_upperCI if Sex=="2" & age=="65+", label(namevar==Study) graphregion(color(white)) name(g7,replace) random title( Anxiety and Depression by female aged 65+(mild) ) wgt(sum_n)

metan additional additional_lowerCI additional_upperCI if Sex=="2" & age=="0-65+", label(namevar==Study) graphregion(color(white)) name(g8,replace) random title( Anxiety and Depression by female aged 0-65+(mild) ) wgt(sum_n)

*severe

metan severe_additional severe_additional_lowerCI severe_additional_upperCI if Sex=="1" & age=="0-18" , label(namevar==Study) graphregion(color(white)) name(g1,replace) random title( Anxiety and Depression by male aged 0-18(severe) ) wgt(sum_n)

metan severe_additional severe_additional_lowerCI severe_additional_upperCI if Sex=="1" & age=="19-64", label(namevar==Study) graphregion(color(white)) name(g2,replace) random title( Anxiety and Depression by male aged 19-64(severe) ) wgt(sum_n)

metan severe_additional severe_additional_lowerCI severe_additional_upperCI if Sex=="1" & age=="65+", label(namevar==Study) graphregion(color(white)) name(g3,replace) random title( Anxiety and Depression by male aged 65+(severe) ) wgt(sum_n)

metan severe_additional severe_additional_lowerCI severe_additional_upperCI if Sex=="1" & age=="0-65+", label(namevar==Study) graphregion(color(white)) name(g4,replace) random title( Anxiety and Depression by male aged 0-65+(severe) ) wgt(sum_n)

metan severe_additional severe_additional_lowerCI severe_additional_upperCI if Sex=="2" & age=="0-18" , label(namevar==Study) graphregion(color(white)) name(g5,replace) random title( Anxiety and Depression by female aged 0-18(severe) ) wgt(sum_n)

metan severe_additional severe_additional_lowerCI severe_additional_upperCI if Sex=="2" & age=="19-64", label(namevar==Study) graphregion(color(white)) name(g6,replace) random title(Anxiety and Depression by female aged 19-64(severe) ) wgt(sum_n)

metan severe_additional severe_additional_lowerCI severe_additional_upperCI if Sex=="2" & age=="65+", label(namevar==Study) graphregion(color(white)) name(g7,replace) random title( Anxiety and Depression by female aged 65+(severe) ) wgt(sum_n)

metan severe_additional severe_additional_lowerCI severe_additional_upperCI if Sex=="2" & age=="0-65+", label(namevar==Study) graphregion(color(white)) name(g8,replace) random title( Anxiety and Depression by female aged 0-65+(severe) ) wgt(sum_n)

clear

list Study n_post n_pre

**mild*

*baseline

gen baseline_var =(baseline*(1-baseline))

gen baseline_lowerCI= baseline-(1.96*sqrt(baseline_var)/n_pre)

gen baseline_upperCI= baseline+(1.96*sqrt(baseline_var)/n_pre)

*final

gen final_var = (final*(1-final))

gen final_lowerCI= final-(1.96*sqrt(final_var)/n_post)

gen final_upperCI= final+(1.96*sqrt(final_var)/n_post)

*pool

gen pool_var = ((baseline_var*(n_pre-1))/(n_pre+n_post))+((final_var *(n_post-1))/(n_pre+n_post))

*additional ci

gen additional = final-baseline

gen additional_lowerCI = (final-baseline)-(1.96*sqrt((pool_var)/(n_post+n_pre)))

gen additional_upperCI = (final-baseline)+(1.96*sqrt((pool_var)/(n_post+n_pre)))

*change in %

gen additionalinper = additional*0.1

*severe

*baseline

gen severe_baseline_var =(severe_baseline*(1-severe_baseline))

gen severe_baseline_lowerCI= severe_baseline-(1.96*sqrt(severe_baseline_var)/severe_n_pre)

gen severe_baseline_upperCI= severe_baseline+(1.96*sqrt(severe_baseline_var)/severe_n_pre)

*final

gen severe_final_var = (severe_final*(1-severe_final))

gen severe_final_lowerCI= severe_final-(1.96*sqrt(severe_final_var)/severe_n_post)

gen severe_final_upperCI= severe_final+(1.96*sqrt(severe_final_var)/severe_n_post)

*pool

gen severe_pool_var = ((severe_baseline_var*(severe_n_pre-1))/(severe_n_pre+severe_n_post))+((severe_final_var *(severe_n_post-1))/(severe_n_pre+severe_n_post))

*additional ci

gen severe_additional = severe_final-severe_baseline

gen severe_additional_lowerCI = (severe_final-severe_baseline)-(1.96*sqrt((severe_pool_var)/(severe_n_post+severe_n_pre)))

gen severe_additional_upperCI = (severe_final-severe_baseline)+(1.96*sqrt((severe_pool_var)/(severe_n_post+severe_n_pre)))

*change in %

gen severe_additionalinper = severe_additional*0.1

*sex

* mild

metan additional additional_lowerCI additional_upperCI if Sex=="1" & age=="0-65+" , label(namevar==Study) graphregion(color(white)) name(g1,replace) random title("Depression in male(mild)") wgt(sum_n)

metan additional additional_lowerCI additional_upperCI if Sex=="2" & age=="0-65+", label(namevar==Study) graphregion(color(white)) name(g2,replace) random title( "Depression in female(mild)") wgt(sum_n)

*sex severe

metan severe_additional severe_additional_lowerCI severe_additional_upperCI if Sex=="1" & age=="0-65+" , label(namevar==Study) graphregion(color(white)) name(g1,replace) random title( "Depression in male (severe)") wgt(sum_n)

metan severe_additional severe_additional_lowerCI severe_additional_upperCI if Sex=="2" & age=="0-65+" , label(namevar==Study) graphregion(color(white)) name(g2,replace) random title( "Depression in female (severe)") wgt(sum_n)

*age

*mild

metan additional additional_lowerCI additional_upperCI if Sex=="1_2" & age=="0-18" , wgt(sum_n) label(namevar==Study) graphregion(color(white)) name(g1,replace) random title( "Depression in aged 0-18(mild)")

metan additional additional_lowerCI additional_upperCI if Sex=="1_2" & age=="19-64", wgt(sum_n) label(namevar==Study) graphregion(color(white)) name(g2,replace) random title( "Depression in aged 19-64(mild) ")

metan additional additional_lowerCI additional_upperCI if Sex=="1_2" & age=="65+", wgt(sum_n) label(namevar==Study) graphregion(color(white)) name(g3,replace) random title( "Depression in aged 65+(mild) ")

metan additional additional_lowerCI additional_upperCI if Sex=="1_2" & age=="0-65+", wgt(sum_n) label(namevar==Study) graphregion(color(white)) name(g4,replace) random title(" Depression in aged 0-65+(mild)" )

*severe

metan severe_additional severe_additional_lowerCI severe_additional_upperCI if Sex=="1_2" & age=="0-18" , label(namevar==Study) graphregion(color(white)) name(g1,replace) random title( "Depression in aged 0-18(severe)" ) wgt(sum_n)

metan severe_additional severe_additional_lowerCI severe_additional_upperCI if Sex=="1_2" & age=="19-64", label(namevar==Study) graphregion(color(white)) name(g2,replace) random title( "Depression in aged 19-64(severe)" ) wgt(sum_n)

metan severe_additional severe_additional_lowerCI severe_additional_upperCI if Sex=="1_2" & age=="65+", label(namevar==Study) graphregion(color(white)) name(g3,replace) random title( "Depression in aged 65+(severe) ") wgt(sum_n)

metan severe_additional severe_additional_lowerCI severe_additional_upperCI if Sex=="1_2" & age=="0-65+", label(namevar==Study) graphregion(color(white)) name(g4,replace) random title("Depression in aged 0-65+(severe)" ) wgt(sum_n)

*agexsex

*mild

metan additional additional_lowerCI additional_upperCI if Sex=="1" & age=="0-18" , label(namevar==Study) graphregion(color(white)) name(g1,replace) random title( "Depression in male aged 0-18(mild) ") wgt(sum_n)

metan additional additional_lowerCI additional_upperCI if Sex=="1" & age=="19-64", label(namevar==Study) graphregion(color(white)) name(g2,replace) random title( "Depression in male aged 19-64(mild)" ) wgt(sum_n)

metan additional additional_lowerCI additional_upperCI if Sex=="1" & age=="65+", label(namevar==Study) graphregion(color(white)) name(g3,replace) random title("Depression in male aged 65+(mild) ") wgt(sum_n)

metan additional additional_lowerCI additional_upperCI if Sex=="1" & age=="0-65+", label(namevar==Study) graphregion(color(white)) name(g4,replace) random title( "Depression in male aged 0-65+(mild)" ) wgt(sum_n)

metan additional additional_lowerCI additional_upperCI if Sex=="2" & age=="0-18" , label(namevar==Study) graphregion(color(white)) name(g5,replace) random title( "Depression in female aged 0-18(mild) ") wgt(sum_n)

metan additional additional_lowerCI additional_upperCI if Sex=="2" & age=="19-64", label(namevar==Study) graphregion(color(white)) name(g6,replace) random title( "Depression infemale aged 19-64(mild)" ) wgt(sum_n)

metan additional additional_lowerCI additional_upperCI if Sex=="2" & age=="65+", label(namevar==Study) graphregion(color(white)) name(g7,replace) random title( "Depression in female aged 65+(mild) ") wgt(sum_n)

metan additional additional_lowerCI additional_upperCI if Sex=="2" & age=="0-65+", label(namevar==Study) graphregion(color(white)) name(g8,replace) random title(" Depression in female aged 0-65+(mild) ") wgt(sum_n)

*severe

metan severe_additional severe_additional_lowerCI severe_additional_upperCI if Sex=="1" & age=="0-18" , label(namevar==Study) graphregion(color(white)) name(g1,replace) random title( "Depression in male aged 0-18(severe) ") wgt(sum_n)

metan severe_additional severe_additional_lowerCI severe_additional_upperCI if Sex=="1" & age=="19-64", label(namevar==Study) graphregion(color(white)) name(g2,replace) random title( "Depression in male aged 19-64(severe)" ) wgt(sum_n)

metan severe_additional severe_additional_lowerCI severe_additional_upperCI if Sex=="1" & age=="65+", label(namevar==Study) graphregion(color(white)) name(g3,replace) random title( "Depression in male aged 65+(severe) ") wgt(sum_n)

metan severe_additional severe_additional_lowerCI severe_additional_upperCI if Sex=="1" & age=="0-65+", label(namevar==Study) graphregion(color(white)) name(g4,replace) random title( "Depression in male aged 0-65+(severe)" ) wgt(sum_n)

metan severe_additional severe_additional_lowerCI severe_additional_upperCI if Sex=="2" & age=="0-18" , label(namevar==Study) graphregion(color(white)) name(g5,replace) random title( "Depression in female aged 0-18(severe) ") wgt(sum_n)

metan severe_additional severe_additional_lowerCI severe_additional_upperCI if Sex=="2" & age=="19-64", label(namevar==Study) graphregion(color(white)) name(g6,replace) random title("Depression in female aged 19-64(severe) ") wgt(sum_n)

metan severe_additional severe_additional_lowerCI severe_additional_upperCI if Sex=="2" & age=="65+", label(namevar==Study) graphregion(color(white)) name(g7,replace) random title( "Depression in female aged 65+(severe) ") wgt(sum_n)

metan severe_additional severe_additional_lowerCI severe_additional_upperCI if Sex=="2" & age=="0-65+", label(namevar==Study) graphregion(color(white)) name(g8,replace) random title( "Depression in female aged 0-65+(severe)" ) wgt(sum_n)

clear

**mild*

*baseline

gen baseline_var =(baseline*(1-baseline))

gen baseline_lowerCI= baseline-(1.96*sqrt(baseline_var)/n_pre)

gen baseline_upperCI= baseline+(1.96*sqrt(baseline_var)/n_pre)

*final

gen final_var = (final*(1-final))

gen final_lowerCI= final-(1.96*sqrt(final_var)/n_post)

gen final_upperCI= final+(1.96*sqrt(final_var)/n_post)

*pool

gen pool_var = ((baseline_var*(n_pre-1))/(n_pre+n_post))+((final_var *(n_post-1))/(n_pre+n_post))

*additional ci

gen additional = final-baseline

gen additional_lowerCI = (final-baseline)-(1.96*sqrt((pool_var)/(n_post+n_pre)))

gen additional_upperCI = (final-baseline)+(1.96*sqrt((pool_var)/(n_post+n_pre)))

*change in %

gen additionalinper = additional*0.1

*severe

*baseline

gen severe_baseline_var =(severe_baseline*(1-severe_baseline))

gen severe_baseline_lowerCI= severe_baseline-(1.96*sqrt(severe_baseline_var)/severe_n_pre)

gen severe_baseline_upperCI= severe_baseline+(1.96*sqrt(severe_baseline_var)/severe_n_pre)

*final

gen severe_final_var = (severe_final*(1-severe_final))

gen severe_final_lowerCI= severe_final-(1.96*sqrt(severe_final_var)/severe_n_post)

gen severe_final_upperCI= severe_final+(1.96*sqrt(severe_final_var)/severe_n_post)

*pool

gen severe_pool_var = ((severe_baseline_var*(severe_n_pre-1))/(severe_n_pre+severe_n_post))+((severe_final_var *(severe_n_post-1))/(severe_n_pre+severe_n_post))

*additional ci

gen severe_additional = severe_final-severe_baseline

gen severe_additional_lowerCI = (severe_final-severe_baseline)-(1.96*sqrt((severe_pool_var)/(severe_n_post+severe_n_pre)))

gen severe_additional_upperCI = (severe_final-severe_baseline)+(1.96*sqrt((severe_pool_var)/(severe_n_post+severe_n_pre)))

*change in %

gen severe_additionalinper = severe_additional*0.1

*sex

* mild

metan additional additional_lowerCI additional_upperCI if Sex=="1" & age=="0-65+" , label(namevar==Study) graphregion(color(white)) name(g1,replace) random title("Anxiety in male(mild)") wgt(sum_n)

metan additional additional_lowerCI additional_upperCI if Sex=="2" & age=="0-65+", label(namevar==Study) graphregion(color(white)) name(g2,replace) random title( "Anxiety in female(mild)") wgt(sum_n)

*sex severe

metan severe_additional severe_additional_lowerCI severe_additional_upperCI if Sex=="1" & age=="0-65+" , label(namevar==Study) graphregion(color(white)) name(g1,replace) random title( "Anxiety in male (severe)") wgt(sum_n)

metan severe_additional severe_additional_lowerCI severe_additional_upperCI if Sex=="2" & age=="0-65+" , label(namevar==Study) graphregion(color(white)) name(g2,replace) random title( "Anxiety in female (severe)") wgt(sum_n)

*age

*mild

metan additional additional_lowerCI additional_upperCI if Sex=="1_2" & age=="0-18" , label(namevar==Study) graphregion(color(white)) name(g1,replace) random title( "Anxiety in aged 0-18(mild)") wgt(sum_n)

metan additional additional_lowerCI additional_upperCI if Sex=="1_2" & age=="19-64", label(namevar==Study) graphregion(color(white)) name(g2,replace) random title( "Anxiety in aged 19-64(mild) ") wgt(sum_n)

metan additional additional_lowerCI additional_upperCI if Sex=="1_2" & age=="65+", label(namevar==Study) graphregion(color(white)) name(g3,replace) random title( "Anxiety in aged 65+(mild) ") wgt(sum_n)

metan additional additional_lowerCI additional_upperCI if Sex=="1_2" & age=="0-65+", label(namevar==Study) graphregion(color(white)) name(g4,replace) random title(" Anxiety in aged 0-65+(mild)" ) wgt(sum_n)

*severe

metan severe_additional severe_additional_lowerCI severe_additional_upperCI if Sex=="1_2" & age=="0-18" , label(namevar==Study) graphregion(color(white)) name(g1,replace) random title( "Anxiety in aged 0-18(severe)" ) wgt(sum_n)

metan severe_additional severe_additional_lowerCI severe_additional_upperCI if Sex=="1_2" & age=="19-64", label(namevar==Study) graphregion(color(white)) name(g2,replace) random title( "Anxiety in aged 19-64(severe)" ) wgt(sum_n)

metan severe_additional severe_additional_lowerCI severe_additional_upperCI if Sex=="1_2" & age=="65+", label(namevar==Study) graphregion(color(white)) name(g3,replace) random title( "Anxiety in aged 65+(severe) ") wgt(sum_n)

metan severe_additional severe_additional_lowerCI severe_additional_upperCI if Sex=="1_2" & age=="0-65+", label(namevar==Study) graphregion(color(white)) name(g4,replace) random title("Anxiety in aged 0-65+(severe)" ) wgt(sum_n)

*agexsex

*mild

metan additional additional_lowerCI additional_upperCI if Sex=="1" & age=="0-18" , label(namevar==Study) graphregion(color(white)) name(g1,replace) random title( "Anxiety in male aged 0-18(mild) ") wgt(sum_n)

metan additional additional_lowerCI additional_upperCI if Sex=="1" & age=="19-64", label(namevar==Study) graphregion(color(white)) name(g2,replace) random title( "Anxiety in male aged 19-64(mild)" ) wgt(sum_n)

metan additional additional_lowerCI additional_upperCI if Sex=="1" & age=="65+", label(namevar==Study) graphregion(color(white)) name(g3,replace) random title("Anxiety in male aged 65+(mild) ") wgt(sum_n)

metan additional additional_lowerCI additional_upperCI if Sex=="1" & age=="0-65+", label(namevar==Study) graphregion(color(white)) name(g4,replace) random title( "Anxiety in male aged 0-65+(mild)" ) wgt(sum_n)

metan additional additional_lowerCI additional_upperCI if Sex=="2" & age=="0-18" , label(namevar==Study) graphregion(color(white)) name(g5,replace) random title( "Anxiety in female aged 0-18(mild) ") wgt(sum_n)

metan additional additional_lowerCI additional_upperCI if Sex=="2" & age=="19-64", label(namevar==Study) graphregion(color(white)) name(g6,replace) random title( "Anxiety infemale aged 19-64(mild)" ) wgt(sum_n)

metan additional additional_lowerCI additional_upperCI if Sex=="2" & age=="65+", label(namevar==Study) graphregion(color(white)) name(g7,replace) random title( "Anxiety in female aged 65+(mild) ") wgt(sum_n)

metan additional additional_lowerCI additional_upperCI if Sex=="2" & age=="0-65+", label(namevar==Study) graphregion(color(white)) name(g8,replace) random title(" Anxiety in female aged 0-65+(mild) ") wgt(sum_n)

*severe

metan severe_additional severe_additional_lowerCI severe_additional_upperCI if Sex=="1" & age=="0-18" , label(namevar==Study) graphregion(color(white)) name(g1,replace) random title( "Anxiety in male aged 0-18(severe) ") wgt(sum_n)

metan severe_additional severe_additional_lowerCI severe_additional_upperCI if Sex=="1" & age=="19-64", label(namevar==Study) graphregion(color(white)) name(g2,replace) random title( "Anxiety in male aged 19-64(severe)" ) wgt(sum_n)

metan severe_additional severe_additional_lowerCI severe_additional_upperCI if Sex=="1" & age=="65+", label(namevar==Study) graphregion(color(white)) name(g3,replace) random title( "Anxiety in male aged 65+(severe) ") wgt(sum_n)

metan severe_additional severe_additional_lowerCI severe_additional_upperCI if Sex=="1" & age=="0-65+", label(namevar==Study) graphregion(color(white)) name(g4,replace) random title( "Anxiety in male aged 0-65+(severe)" ) wgt(sum_n)

metan severe_additional severe_additional_lowerCI severe_additional_upperCI if Sex=="2" & age=="0-18" , label(namevar==Study) graphregion(color(white)) name(g5,replace) random title( "Anxiety in female aged 0-18(severe) ") wgt(sum_n)

metan severe_additional severe_additional_lowerCI severe_additional_upperCI if Sex=="2" & age=="19-64", label(namevar==Study) graphregion(color(white)) name(g6,replace) random title("Anxiety in female aged 19-64(severe) ") wgt(sum_n)

metan severe_additional severe_additional_lowerCI severe_additional_upperCI if Sex=="2" & age=="65+", label(namevar==Study) graphregion(color(white)) name(g7,replace) random title( "Anxiety in female aged 65+(severe) ") wgt(sum_n)

metan severe_additional severe_additional_lowerCI severe_additional_upperCI if Sex=="2" & age=="0-65+", label(namevar==Study) graphregion(color(white)) name(g8,replace) random title( "Anxiety in female aged 0-65+(severe)" ) wgt(sum_n)

clear

*rq3

**mild*

*baseline

gen baseline_var =(baseline*(1-baseline))

gen baseline_lowerCI= baseline-(1.96*sqrt(baseline_var)/n_pre)

gen baseline_upperCI= baseline+(1.96*sqrt(baseline_var)/n_pre)

*final

gen final_var = (final*(1-final))

gen final_lowerCI= final-(1.96*sqrt(final_var)/n_post)

gen final_upperCI= final+(1.96*sqrt(final_var)/n_post)

*pool

gen pool_var = ((baseline_var*(n_pre-1))/(n_pre+n_post))+((final_var *(n_post-1))/(n_pre+n_post))

*additional ci

gen additional = final-baseline

gen additional_lowerCI = (final-baseline)-(1.96*sqrt((pool_var)/(n_post+n_pre)))

gen additional_upperCI = (final-baseline)+(1.96*sqrt((pool_var)/(n_post+n_pre)))

*change in %

gen additionalinper = additional*0.1

metan additional additional_lowerCI additional_upperCI, wgt(sum_n) label(namevar==Study) random title(Anxiety and Depression (mild))

metareg additional schoolrestriction sum_n, random wsse(_seES)

metareg additional socialdistancing sum_n, random wsse(_seES)

/*

metareg additional femaleratio, random wsse(_seES)

metareg additional new_cases, random wsse(_seES)

metareg additional total_deaths, random wsse(_seES)

metareg additional new_deaths, random wsse(_seES)

metareg additional total_deaths_per_million, random wsse(_seES)

metareg additional new_deaths_per_million , random wsse(_seES)

metareg additional positive_rate , random wsse(_seES)

metareg additional population , random wsse(_seES)

metareg additional gdp_per_capita , random wsse(_seES)

metareg additional Accdeathpercapita , random wsse(_seES)

metareg additional Newdeathpercapita, random wsse(_seES)

metareg additional Newcasepercapita, random wsse(_seES)

metareg additional schoolrestriction socialdistancing, random wsse(_seES)

metareg additional schoolrestriction socialdistancing gdp_per_capita population , random wsse(_seES)

metareg additional schoolrestriction gdp_per_capita population , random wsse(_seES)

metareg additional socialdistancing gdp_per_capita population , random wsse(_seES)

*/

clear

*Rq3

*severe

*baseline

gen severe_baseline_var =(severe_baseline*(1-severe_baseline))

gen severe_baseline_lowerCI= severe_baseline-(1.96*sqrt(severe_baseline_var)/severe_n_pre)

gen severe_baseline_upperCI= severe_baseline+(1.96*sqrt(severe_baseline_var)/severe_n_pre)

*final

gen severe_final_var = (severe_final*(1-severe_final))

gen severe_final_lowerCI= severe_final-(1.96*sqrt(severe_final_var)/severe_n_post)

gen severe_final_upperCI= severe_final+(1.96*sqrt(severe_final_var)/severe_n_post)

*pool

gen severe_pool_var = ((severe_baseline_var*(severe_n_pre-1))/(severe_n_pre+severe_n_post))+((severe_final_var *(severe_n_post-1))/(severe_n_pre+severe_n_post))

*additional ci

gen severe_additional = severe_final-severe_baseline

gen severe_additional_lowerCI = (severe_final-severe_baseline)-(1.96*sqrt((severe_pool_var)/(severe_n_post+severe_n_pre)))

gen severe_additional_upperCI = (severe_final-severe_baseline)+(1.96*sqrt((severe_pool_var)/(severe_n_post+severe_n_pre)))

*change in %

gen severe_additionalinper = severe_additional*0.1

metan severe_additional severe_additional_lowerCI severe_additional_upperCI, wgt(sum_n) label(namevar==Study) random title(Anxiety and Depression (severe))

metareg severe_additional schoolrestriction sum_n, random wsse(_seES)

metareg severe_additional socialdistancing sum_n, random wsse(_seES)

/*

metareg additional femaleratio, random wsse(_seES)

metareg additional new_cases, random wsse(_seES)

metareg additional total_deaths, random wsse(_seES)

metareg additional new_deaths, random wsse(_seES)

metareg additional total_deaths_per_million, random wsse(_seES)

metareg additional new_deaths_per_million , random wsse(_seES)

metareg additional positive_rate , random wsse(_seES)

metareg additional population , random wsse(_seES)

metareg additional gdp_per_capita , random wsse(_seES)

metareg additional Accdeathpercapita , random wsse(_seES)

metareg additional Newdeathpercapita, random wsse(_seES)

metareg additional Newcasepercapita, random wsse(_seES)

metareg additional schoolrestriction socialdistancing, random wsse(_seES)

metareg additional schoolrestriction socialdistancing gdp_per_capita population , random wsse(_seES)

metareg additional schoolrestriction gdp_per_capita population , random wsse(_seES)

metareg additional socialdistancing gdp_per_capita population , random wsse(_seES)

*/clear

*rq3

**mild*

*baseline

gen baseline_var =(baseline*(1-baseline))

gen baseline_lowerCI= baseline-(1.96*sqrt(baseline_var)/n_pre)

gen baseline_upperCI= baseline+(1.96*sqrt(baseline_var)/n_pre)

*final

gen final_var = (final*(1-final))

gen final_lowerCI= final-(1.96*sqrt(final_var)/n_post)

gen final_upperCI= final+(1.96*sqrt(final_var)/n_post)

*pool

gen pool_var = ((baseline_var*(n_pre-1))/(n_pre+n_post))+((final_var *(n_post-1))/(n_pre+n_post))

*additional ci

gen additional = final-baseline

gen additional_lowerCI = (final-baseline)-(1.96*sqrt((pool_var)/(n_post+n_pre)))

gen additional_upperCI = (final-baseline)+(1.96*sqrt((pool_var)/(n_post+n_pre)))

*change in %

gen additionalinper = additional*0.1

metan additional additional_lowerCI additional_upperCI, wgt(sum_n) label(namevar==Study) random title(Anxiety and Depression (mild))

metareg additional schoolrestriction, random wsse(_seES)

metareg additional socialdistancing, random wsse(_seES)

metareg additional femaleratio, random wsse(_seES)

metareg additional new_cases, random wsse(_seES)

metareg additional total_deaths, random wsse(_seES)

metareg additional new_deaths, random wsse(_seES)

metareg additional total_deaths_per_million, random wsse(_seES)

metareg additional new_deaths_per_million , random wsse(_seES)

metareg additional positive_rate , random wsse(_seES)

metareg additional population , random wsse(_seES)

metareg additional gdp_per_capita , random wsse(_seES)

metareg additional Accdeathpercapita , random wsse(_seES)

metareg additional Newdeathpercapita, random wsse(_seES)

metareg additional Newcasepercapita, random wsse(_seES)

metareg additional schoolrestriction socialdistancing, random wsse(_seES)

metareg additional schoolrestriction socialdistancing gdp_per_capita population , random wsse(_seES)

metareg additional schoolrestriction gdp_per_capita population , random wsse(_seES)

metareg additional socialdistancing gdp_per_capita population , random wsse(_seES)

clear

*Rq3

*severe

*baseline

gen severe_baseline_var =(severe_baseline*(1-severe_baseline))

gen severe_baseline_lowerCI= severe_baseline-(1.96*sqrt(severe_baseline_var)/severe_n_pre)

gen severe_baseline_upperCI= severe_baseline+(1.96*sqrt(severe_baseline_var)/severe_n_pre)

*final

gen severe_final_var = (severe_final*(1-severe_final))

gen severe_final_lowerCI= severe_final-(1.96*sqrt(severe_final_var)/severe_n_post)

gen severe_final_upperCI= severe_final+(1.96*sqrt(severe_final_var)/severe_n_post)

*pool

gen severe_pool_var = ((severe_baseline_var*(severe_n_pre-1))/(severe_n_pre+severe_n_post))+((severe_final_var *(severe_n_post-1))/(severe_n_pre+severe_n_post))

*additional ci

gen severe_additional = severe_final-severe_baseline

gen severe_additional_lowerCI = (severe_final-severe_baseline)-(1.96*sqrt((severe_pool_var)/(severe_n_post+severe_n_pre)))

gen severe_additional_upperCI = (severe_final-severe_baseline)+(1.96*sqrt((severe_pool_var)/(severe_n_post+severe_n_pre)))

*change in %

gen severe_additionalinper = severe_additional*0.1

metan severe_additional severe_additional_lowerCI severe_additional_upperCI, wgt(sum_n) label(namevar==Study) random title(Anxiety and Depression (severe))

metareg severe_additional schoolrestriction, random wsse(_seES)

metareg severe_additional socialdistancing, random wsse(_seES)

metareg severe_additional femaleratio, random wsse(_seES)

metareg severe_additional new_cases, random wsse(_seES)

metareg severe_additional total_deaths, random wsse(_seES)

metareg severe_additional new_deaths, random wsse(_seES)

metareg severe_additional total_deaths_per_million, random wsse(_seES)

metareg severe_additional new_deaths_per_million , random wsse(_seES)

metareg severe_additional positive_rate , random wsse(_seES)

metareg severe_additional population , random wsse(_seES)

metareg severe_additional gdp_per_capita , random wsse(_seES)

metareg severe_additional Accdeathpercapita , random wsse(_seES)

metareg severe_additional Newdeathpercapita, random wsse(_seES)

metareg severe_additional Newcasepercapita, random wsse(_seES)

metareg severe_additional schoolrestriction socialdistancing, random wsse(_seES)

metareg severe_additional schoolrestriction socialdistancing gdp_per_capita population , random wsse(_seES)

metareg severe_additional schoolrestriction gdp_per_capita population , random wsse(_seES)

metareg severe_additional socialdistancing gdp_per_capita population , random wsse(_seES)

clear

*rq3

**mild*

*baseline

gen baseline_var =(baseline*(1-baseline))

gen baseline_lowerCI= baseline-(1.96*sqrt(baseline_var)/n_pre)

gen baseline_upperCI= baseline+(1.96*sqrt(baseline_var)/n_pre)

*final

gen final_var = (final*(1-final))

gen final_lowerCI= final-(1.96*sqrt(final_var)/n_post)

gen final_upperCI= final+(1.96*sqrt(final_var)/n_post)

*pool

gen pool_var = ((baseline_var*(n_pre-1))/(n_pre+n_post))+((final_var *(n_post-1))/(n_pre+n_post))

*additional ci

gen additional = final-baseline

gen additional_lowerCI = (final-baseline)-(1.96*sqrt((pool_var)/(n_post+n_pre)))

gen additional_upperCI = (final-baseline)+(1.96*sqrt((pool_var)/(n_post+n_pre)))

*change in %

gen additionalinper = additional*0.1

metan additional additional_lowerCI additional_upperCI, wgt(sum_n) label(namevar==Study) random title(Anxiety and Depression (mild))

metareg additional schoolrestriction, random wsse(_seES)

metareg additional socialdistancing, random wsse(_seES)

metareg additional femaleratio, random wsse(_seES)

metareg additional new_cases, random wsse(_seES)

metareg additional total_deaths, random wsse(_seES)

metareg additional new_deaths, random wsse(_seES)

metareg additional total_deaths_per_million, random wsse(_seES)

metareg additional new_deaths_per_million , random wsse(_seES)

metareg additional positive_rate , random wsse(_seES)

metareg additional population , random wsse(_seES)

metareg additional gdp_per_capita , random wsse(_seES)

metareg additional Accdeathpercapita , random wsse(_seES)

metareg additional Newdeathpercapita, random wsse(_seES)

metareg additional Newcasepercapita, random wsse(_seES)

metareg additional schoolrestriction socialdistancing, random wsse(_seES)

metareg additional schoolrestriction socialdistancing gdp_per_capita population , random wsse(_seES)

metareg additional schoolrestriction gdp_per_capita population , random wsse(_seES)

metareg additional socialdistancing gdp_per_capita population , random wsse(_seES)

clear

*Rq3

*severe

*baseline

gen severe_baseline_var =(severe_baseline*(1-severe_baseline))

gen severe_baseline_lowerCI= severe_baseline-(1.96*sqrt(severe_baseline_var)/severe_n_pre)

gen severe_baseline_upperCI= severe_baseline+(1.96*sqrt(severe_baseline_var)/severe_n_pre)

*final

gen severe_final_var = (severe_final*(1-severe_final))

gen severe_final_lowerCI= severe_final-(1.96*sqrt(severe_final_var)/severe_n_post)

gen severe_final_upperCI= severe_final+(1.96*sqrt(severe_final_var)/severe_n_post)

*pool

gen severe_pool_var = ((severe_baseline_var*(severe_n_pre-1))/(severe_n_pre+severe_n_post))+((severe_final_var *(severe_n_post-1))/(severe_n_pre+severe_n_post))

*additional ci

gen severe_additional = severe_final-severe_baseline

gen severe_additional_lowerCI = (severe_final-severe_baseline)-(1.96*sqrt((severe_pool_var)/(severe_n_post+severe_n_pre)))

gen severe_additional_upperCI = (severe_final-severe_baseline)+(1.96*sqrt((severe_pool_var)/(severe_n_post+severe_n_pre)))

*change in %

gen severe_additionalinper = severe_additional*0.1

metan severe_additional severe_additional_lowerCI severe_additional_upperCI, wgt(sum_n) label(namevar==Study) random title(Anxiety and Depression (severe))

metareg severe_additional schoolrestriction, random wsse(_seES)

metareg severe_additional socialdistancing, random wsse(_seES)

metareg severe_additional femaleratio, random wsse(_seES)

metareg severe_additional new_cases, random wsse(_seES)

metareg severe_additional total_deaths, random wsse(_seES)

metareg severe_additional new_deaths, random wsse(_seES)

metareg severe_additional total_deaths_per_million, random wsse(_seES)

metareg severe_additional new_deaths_per_million , random wsse(_seES)

metareg severe_additional positive_rate , random wsse(_seES)

metareg severe_additional population , random wsse(_seES)

metareg severe_additional gdp_per_capita , random wsse(_seES)

metareg severe_additional Accdeathpercapita , random wsse(_seES)

metareg severe_additional Newdeathpercapita, random wsse(_seES)

metareg severe_additional Newcasepercapita, random wsse(_seES)

metareg severe_additional schoolrestriction socialdistancing, random wsse(_seES)

metareg severe_additional schoolrestriction socialdistancing gdp_per_capita population , random wsse(_seES)

metareg severe_additional schoolrestriction gdp_per_capita population , random wsse(_seES)

metareg severe_additional socialdistancing gdp_per_capita population , random wsse(_seES)

***Additional estimations

**Mild anxiety and depression combined

preserve

keep if additional != .

keep if additional != 0

keep if sex==""

keep if age=="0-65+"

*Prediction interval

meta set additional additional_lowerCI additional_upperCI, random(dlaird)

meta summarize, wgt(sum_n) predint

*Egger’s test and funnel plot

meta bias, egger

meta funnelplot, title("Mild depression and anxiety combined")

*Trim-and-fill analysis

gen SE_additional =( ( additional_upperCI ) - ( additional_lowerCI )) / (2*invnorm(.975))

metan additional additional_lowerCI additional_upperCI, wgt(sum_n)

metatrim additional SE_additional, funnel print

*Influence (leave-one-out) analysis

metan additional additional_lowerCI additional_upperCI, random wgt(sum_n) study(Study)

metaninf additional additional_lowerCI additional_upperCI, random wgt(sum_n) label(namevar=Study)

*Meta-regression with additional adjustment for study quality

metan additional additional_lowerCI additional_upperCI, random wgt(sum_n) study(Study)

metareg additional schoolrestriction sum_n, random wsse(_seES)

metareg additional schoolrestriction sum_n Studyqual, random wsse(_seES)

restore

**Mild anxiety alone

preserve

drop if Outcome!="Anxiety"

keep if additional != .

keep if additional != 0

keep if sex==""

keep if age=="0-65+"

*Prediction interval

meta set additional additional_lowerCI additional_upperCI, random(dlaird)

meta summarize, wgt(sum_n) predint

*Egger’s test and funnel plot

meta bias, egger

meta funnelplot, title("Mild anxiety alone")

*Trim-and-fill analysis

gen SE_additional =( ( additional_upperCI ) - ( additional_lowerCI )) / (2*invnorm(.975))

metan additional additional_lowerCI additional_upperCI, wgt(sum_n)

metatrim additional SE_additional, funnel print

*Influence (leave-one-out) analysis

metan additional additional_lowerCI additional_upperCI, random wgt(sum_n) study(Study)

metaninf additional additional_lowerCI additional_upperCI, random wgt(sum_n) label(namevar=Study)

*Meta-regression with additional adjustment for study quality

metan additional additional_lowerCI additional_upperCI, random wgt(sum_n) study(Study)

metareg additional schoolrestriction sum_n, random wsse(_seES)

metareg additional schoolrestriction sum_n Studyqual, random wsse(_seES)

restore

**Mild depression alone

preserve

drop if Outcome!="Depression"

keep if additional != .

keep if additional != 0

keep if sex==""

keep if age=="0-65+"

*Prediction interval

meta set additional additional_lowerCI additional_upperCI, random(dlaird)

meta summarize, wgt(sum_n) predint

*Egger’s test and funnel plot

meta bias, egger

meta funnelplot, title("Mild depression alone")

*Trim-and-fill analysis

gen SE_additional =( ( additional_upperCI ) - ( additional_lowerCI )) / (2*invnorm(.975))

metan additional additional_lowerCI additional_upperCI, wgt(sum_n)

metatrim additional SE_additional, funnel print

*Influence (leave-one-out) analysis

metan additional additional_lowerCI additional_upperCI, random wgt(sum_n) study(Study)

metaninf additional additional_lowerCI additional_upperCI, random wgt(sum_n) label(namevar=Study)

*Meta-regression with additional adjustment for study quality

metan additional additional_lowerCI additional_upperCI, random wgt(sum_n) study(Study)

metareg additional schoolrestriction sum_n, random wsse(_seES)

metareg additional schoolrestriction sum_n Studyqual, random wsse(_seES)

restore

***Additional estimations

**Severe anxiety and depression combined

preserve

keep if severe_additional != .

keep if severe_additional != 0

keep if sex==""

keep if age=="0-65+"

*Prediction interval

meta set severe_additional severe_additional_lowerCI severe_additional_upperCI, random(dlaird)

meta summarize, wgt(sum_n) predint

*Egger’s test and funnel plot

meta bias, egger

meta funnelplot, title("Severe depression and anxiety combined")

*Trim-and-fill analysis

gen SE_severe_additional =( ( severe_additional_upperCI ) - ( severe_additional_lowerCI )) / (2*invnorm(.975))

metan severe_additional severe_additional_lowerCI severe_additional_upperCI, wgt(sum_n)

metatrim severe_additional SE_severe_additional, funnel print

*Influence (leave-one-out) analysis

metan severe_additional severe_additional_lowerCI severe_additional_upperCI, random wgt(sum_n) study(Study)

metaninf severe_additional severe_additional_lowerCI severe_additional_upperCI, random wgt(sum_n) label(namevar=Study)

*Meta-regression with additional adjustment for study quality

metan severe_additional severe_additional_lowerCI severe_additional_upperCI, random wgt(sum_n) study(Study)

metareg severe_additional schoolrestriction sum_n, random wsse(_seES)

metareg severe_additional schoolrestriction sum_n Studyqual, random wsse(_seES)

restore

**Severe anxiety alone

preserve

drop if Outcome!="Anxiety"

keep if additional != .

keep if additional != 0

keep if sex==""

keep if age=="0-65+"

*Prediction interval

meta set severe_additional severe_additional_lowerCI severe_additional_upperCI, random(dlaird)

meta summarize, wgt(sum_n) predint

*Egger’s test and funnel plot

meta bias, egger

meta funnelplot, title("Severe anxiety alone")

*Trim-and-fill analysis

gen SE_severe_additional =( ( severe_additional_upperCI ) - ( severe_additional_lowerCI )) / (2*invnorm(.975))

metan severe_additional severe_additional_lowerCI severe_additional_upperCI, wgt(sum_n)

metatrim severe_additional SE_severe_additional, funnel print

*Influence (leave-one-out) analysis

metan severe_additional severe_additional_lowerCI severe_additional_upperCI, random wgt(sum_n) study(Study)

metaninf severe_additional severe_additional_lowerCI severe_additional_upperCI, random wgt(sum_n) label(namevar=Study)

*Meta-regression with additional adjustment for study quality

metan severe_additional severe_additional_lowerCI severe_additional_upperCI, random wgt(sum_n) study(Study)

metareg severe_additional schoolrestriction sum_n, random wsse(_seES)

metareg severe_additional schoolrestriction sum_n Studyqual, random wsse(_seES)

restore

**Severe depression alone

preserve

drop if Outcome!="Depression"

keep if severe_additional != .

keep if severe_additional != 0

keep if sex==""

keep if age=="0-65+"

*Prediction interval

meta set severe_additional severe_additional_lowerCI severe_additional_upperCI, random(dlaird)

meta summarize, wgt(sum_n) predint

*Egger’s test and funnel plot

meta bias, egger

meta funnelplot, title("Severe depression alone")

*Trim-and-fill analysis

gen SE_severe_additional =( ( severe_additional_upperCI ) - ( severe_additional_lowerCI )) / (2*invnorm(.975))

metan severe_additional severe_additional_lowerCI severe_additional_upperCI, wgt(sum_n)

metatrim severe_additional SE_severe_additional, funnel print

*Influence (leave-one-out) analysis

metan severe_additional severe_additional_lowerCI severe_additional_upperCI, random wgt(sum_n) study(Study)

metaninf severe_additional severe_additional_lowerCI severe_additional_upperCI, random wgt(sum_n) label(namevar=Study)

*Meta-regression with additional adjustment for study quality

metan severe_additional severe_additional_lowerCI severe_additional_upperCI, random wgt(sum_n) study(Study)

metareg severe_additional schoolrestriction sum_n, random wsse(_seES)

metareg severe_additional schoolrestriction sum_n Studyqual, random wsse(_seES)

restore

log close
